# The Seattle Midlife Women’s Health Study: a longitudinal prospective study of women during the menopausal transition and early postmenopause

**DOI:** 10.1186/s40695-016-0019-x

**Published:** 2016-11-09

**Authors:** Nancy Fugate Woods, Ellen Sullivan Mitchell

**Affiliations:** 1 Department of Biobehavioral Nursing, University of Washington, Seattle, WA 98195 USA; 2 Department of Family and Child Nursing, University of Washington, Seattle, WA 98195, USA

**Keywords:** Menopausal transition, Staging reproductive aging, Menopause, Midlife cohort, Symptoms, Endocrine changes

## Abstract

**Background:**

The need for longitudinal, population-based studies to illuminate women’s experiences of symptoms during the menopausal transition motivated the development of the Seattle Midlife Women’s Health Study.

**Methods:**

Longitudinal, population-based study of symptoms women experienced between the Late Reproductive stage of reproductive aging and the early postmenopause. Data collection began in 1990 with 508 women ages 35–55 and continued to 2013. Entry criteria included age, at least one period in past 12 months, uterus intact and at least 1 ovary. Women were studied up to 5 years postmenopause. Data collection included yearly health questionnaires, health diaries, urinary hormonal assays, menstrual calendars and buccal cell smears.

**Results:**

Contributions of the study included development of a method for staging the menopausal transition; development of bleeding criteria to differentiate bleeding episodes from intermenstrual bleeding from menstrual calendars; identification of hormonal changes associated with menopausal transition stages; assessment of the effects of menopausal transition factors, aging, stress-related factors, health factors, social factors on symptoms, particularly hot flashes, depressed mood, pain, cognitive, sexual desire, and sleep disruption symptoms, and urinary incontinence symptoms; identification of naturally occurring clusters of symptoms women experienced during the menopausal transition and early postmenopause; and assessment of gene polymorphisms associated with events such as onset of the early and late menopausal transition stages and symptoms.

**Conclusions:**

Over the course of the longitudinal Seattle Midlife Women's Health Study, investigators contributed to understanding of symptoms women experience during the menopausal transition and early postmenopause as well as methods of staging reproductive aging.

**Electronic supplementary material:**

The online version of this article (doi:10.1186/s40695-016-0019-x) contains supplementary material, which is available to authorized users.

## Background

During the 1970s and 1980s attention to women’s health research increased in the US, culminating in several important milestones, among them establishment of the Office of Women’s Health Research in the National Institutes of Health in 1991 and development of the first US Women’s Health Research Agenda [1]. In 1993 the National Institutes of Health/National Institute on Aging, National Institute of Child Health and Development, and collaborating organizations convened a workshop on Menopause to provide focus for future research about midlife women and menopause. This work was preceded by the landmark longitudinal study of the menopausal transition (MT): the Massachusetts Women’s Health Study begun in 1982 [2], a longitudinal study developed to expand knowledge about the experiences of a community-based population of women as they traversed the MT. This focus on a community-based population was in contrast to earlier studies of clinical populations. Another early effort by Matthews and colleagues recruited women from the state of Pennsylvania (The Healthy Women Study) to determine the natural history of the MT, and behavioral and biological changes that occurred during the MT and postmenopause (PM) and their effects on cardiovascular disease risk [3].

The Seattle Midlife Women’s Health Study (SMWHS) was built on the foundational studies of the 1980s, including our longitudinal studies of perimenstrual symptoms that focused on women after they had reached age 40 years [4–6]. The SMWHS originated as one component of a Center for Women’s Health Research (CWHR) at the University of Washington School of Nursing in 1989. Funded by the National Institute of Nursing Research, the CWHR was created by an interdisciplinary cadre of investigators to support research development in women’s health [7].

### Overview of the SMWHS

The initial phase of SMWHS from September 1989 to July 1996, as part of the CWHR, was designed to test a model relating MT status, stress exposure, socialization for midlife, personal and social factors modulating midlife experiences, reproductive health history, and health behaviors to health status and health-seeking behavior in midlife women between the ages of 35 and 55. A model including menopausal changes, socialization for midlife, health status, stressful life context, and vasomotor symptoms guided analysis of depressed mood symptoms, an outcome of interest [8]. The model was generalized to other symptoms, for example, by testing outcomes including hot flashes, sleep, cognitive, mood, and pain symptoms, to name a few. (The Aims for Phase 1 are included in Table 1)

**Table 1:** Aims for the Seattle Midlife Women’s Health Study by Phase

**Phase 1: 1990–1996**
The aim of Phase I was to test a model relating menopausal status, stress exposure, socialization for midlife, personal and social factors modulating midlife experiences, reproductive health history, and health behaviors to health status and health-seeking behavior in midlife women between the ages of 35 and 55.
**Phase 2: 1996–2001**
**Aim 1.** Describe the **progression through stages of the perimenopuse** (pre transition, early transition, middle transition, late transition, and postmenopause as determined from annual health updates and daily menstrual calendars) for women over a nine year period with respect to: a) **Symptoms**, including vasomotor, dysphoric mood, insomnia, somatic, and discomfort symptoms, recorded in a daily health diary for three days monthly (coinciding with hormone assays); b) **Altered ovarian function** (estrone, testosterone (T), and FSH), measured in first morning urine samples at monthly intervals; c) **perceived stress** (stressful life events, income inadequacy) measured annually and perceived stress measured 3 days each month in the health diary; d) **stress arousal** (urinary levels of cortisol and catecholamines) measured in the first morning urine samples at monthly intervals; and e)** symptom management**, including use of health services and hormone replacement therapy assessed annually in a health update questionnaire and interview. **Aim 2.** Test the following **hypotheses regarding symptoms** during the three stages of the transition to menopause (early to middle to late): a) women who experience more severe **vasomotor symptoms** during the transition to menopause will have: higher levels of perceived stress, lower levels of estrone, and higher levels of catecholamines and cortisol; b) women who experience more severe **dysphoric mood symptoms** during the transition to menopause will have: higher levels of perceived stress, higher levels of cortisol, and norepinepherine, and a lower estrogen:androgen ratio; c) women who experience more sever **insomnia symptoms** during the transition to menopause will have: higher levels of perceived stress, lower levels of estrone, and higher levels of catecholamines. **Aim 3.** Test the relationship within individual women among HPO axis hormones (estrone, FSH, testosterone), indicators of physiologic stress arousal (cortisol and catecholamines), daily stress ratings, and symptoms (especially vasomotor, dysphoric mood, and insomnia), measured monthly over a nine year period, using auto-correlation and cross-correlation techniques. **Aim 4.** To estimate the stability of symptom patterns women have recorded in daily health diaries each year with symptom patterns women experience during the menopausal transition (over the period of 1991 to 1995, 1996–2000 and 2001–2005).
**Phase 3: 2002–2006**
**Aim 1**. Describe and compare women in the menopausal transition (early, middle and late transition), in the early postmenopause, and those who use HRT, on indicators of pituitary-ovarian hormone changes, perceived stress, physiologic stress arousal, vasomotor, dysphoric mood, somatic, discomfort and insomnia symptoms. ***Aim 1 Hypotheses:*** Hypothesis 1: Women in late transition will have higher levels of urinary FSH, cortisol and norepinepherine, higher perceived stress and higher vasomotor symptom severity than women in early or middle transition. Hypothesis 2: Women in the postmenopause will have lower levels of urinary estrone and testosterone, lower perceived stress and higher levels of FSH and vasomotor symptoms than women in the three menopausal transition stages. Hypothesis 3: There will be no group differences among women in the three menopausal transition stages for urinary estrone, testosterone and epinephrine, depressed mood or the 5 symptom clusters except for vasomotor symptoms. Hypothesis 4: Women on HRT will have higher estrone levels and lower perceived stress, urinary cortisol, and vasomotor symptoms than women who are not on HRT, those in the menopausal transition or those who are postmenopausal. **Aim 2**. Compare women in the menopausal transition and early postmenopause with different *estrogen metabolism and catabolism gene polymorphisms* with respect to estradiol and estrone levels, age of onset of middle and late menopausal transition stage and menopause, and heaviness of menstrual blood flow. **Aim 3.** Compare women in the menopausal transition and early postmenopause with different *estrogen receptor gene polymorphisms* with respect to estradiol and estrone levels, age of onset of middle and late menopausal transition stage and menopause, and heaviness of menstrual blood flow.
**Phase 4: 2007–2013**
Continuation of aims from Phases 1 –3. Additional aims for the Symptom Cluster Study that was part of Phase 4. 1. Identify symptom clusters (SC) SMWHS participants experienced during the late reproductive, early menopausal transition stages and early postmenopause using latent class analysis to complement the preliminary analyses of the late stage SCs; 2. Determine the consistency of SCs with the clusters identified for the late menopausal transition stage across the late reproductive stage, early menopausal transition stage and early postmenopause; 3. Test models hypothesizing the relationship between SC groups and profiles of: a) gene polymorphisms in the estrogen synthesis pathways (CYP 19 and 17 HSD) and genes polymorphisms in neuroendocrine pathways modulated by estrogen (5HTTLPR, NPY, BDNF); b) hypothalamic-pituitary-ovarian (HPO) biomarkers (E, T, FSH), and hypothalamic-pituitary-adrenal (HPA – cortisol) and autonomic nervous system (ANS- epinephrine, norepinephrine) biomarkers; c) reproductive aging stages (late reproductive, early and late menopausal transition, and early postmenopause); d) socio-behavioral risk factors (e.g. high stress, role burden, low income adequacy, employment, education, social support); e) symptom vulnerability factors (e.g. history of sexual abuse, low mastery, self-consciousness, low self esteem); and outcomes of well-being and interference with work and relationships; 4. Based on a systematic review of controlled clinical trials for managing hot flashes, identify treatment effects on co-occuring symptoms and reported adverse treatment effects, including sleep disturbances, mood, pain and cognitive symptoms; 5. Synthesize results of the empirical analyses (aims 1–3) and systematic review (aim 4) to develop novel symptom cluster management protocols to be tested in a future feasibility study. Additional aims for Urinary Incontinence Study that was part of Phase 4. 1. Determine the influence of age and menopausal transition factors on the experience of urinary incontinence (stress, urge and any incontinence) among midlife women; 2. Assess the influence of lifespan health factors and life context (personal and social resources and stress) on urinary incontinence; and 3. Determine the relationship between urinary incontinence and well-being, symptoms (fatigue, disrupted sleep, anxiety and depressed mood) and interference with daily living (work and relationships).

Based on results of the initial study, additional funding was obtained for the SMWHS from July 1996 to February 2001. This second funding phase focused on the MT and its relationship to symptoms, altered ovarian function, perceived stress and stress arousal, as well as symptom management. A major addition to the measures for this study included hormonal assays (urinary estrone, FSH, testosterone, cortisol and catecholamines). (Aims for Phase 2 are included in Table 1).

A third funding phase spanned 2002 through 2006. Phase 3 of SMWHS continued to focus on symptoms as the primary endpoints. In addition to linking the symptoms to endocrine patterns, the effect of gene polymorphisms in estrogen synthesis, metabolism and receptor genes was added to the aims. (Detailed aims for Phase 3 are given in Table 1).

**Fig. 1:**
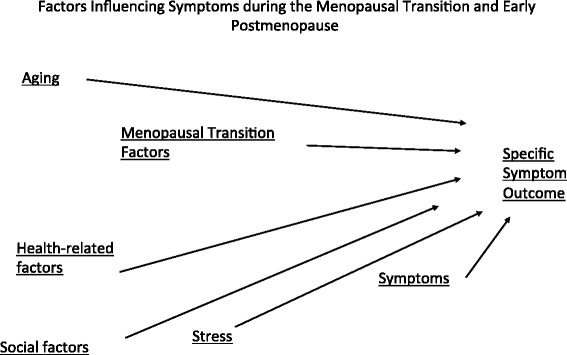
General Model Guiding SMWHS Symptom Analyses Across Time

A fourth and final phase of data collection, after the end of major funding, continued from 2007 to 2013. The focus of this phase was to complete the data collection for the study. Women who had not yet reached 5 years PM, were not taking any estrogen and had an intact uterus were entered into this final phase. Aims for this phase were a combination of the aims for the three prior phases of funding. The model guiding the longitudinal analysis of symptom data across all 4 phases is depicted in Fig. 1.

Two small grants supported the fourth phase of the study. The first was “Menopausal Transition Symptom Clusters: Genetic, Endocrine, and Social Correlates” that focused on the secondary analyses of symptom data, particularly on multiple co-occurring symptoms called symptom clusters that women experienced during the MT and early PM. Symptom data were analyzed to identify clusters of symptoms women experienced and to relate them to stress, health behaviors, health status, endocrine patterns, and gene polymorphisms. (Aims for this study are in Table 1). A second small grant during the fourth phase of the study, Urinary Incontinence during the Menopausal Transition and Early Postmenopause, was awarded by Pfizer, Inc, Medical Division, that supported the secondary analysis of urinary incontinence data over time. (Aims for this study are in Table 1).

In addition, research support was provided by intramural funds to develop a scannable health diary form, for a pilot study of gene polymorphisms related to symptoms, and to complete collection of data from women as they experienced the early PM (Research Intramural Funding Program, University of Washington School of Nursing).

## Methods

### Design

A prospective, repeated measures design was used to study a population-based sample of women who were about to begin or had begun the transition to menopause at the time of entry into the study. Data were collected throughout the study at intervals described below for a total of 23 years. The study was divided into 4 phases based on the aims associated with each funding period. Each phase expanded the aims of the previous phase.

### Sample

From early 1990 to early 1992, 508 women were enrolled. This original population-based sample from the Seattle area was obtained by telephone screening of all households in over 20 census tracts selected for mixed ethnicity and mixed income. There were 13,120 households enumerated. Of the 11,222 households able to be contacted (85.5 % of those enumerated), 1,428 women between the ages of 35 and 55 were screened (12.7 % of those contacted) and 820 were eligible (57.4 % of those screened). In addition to age, a woman was eligible if she had an intact uterus and at least one ovary, had at least one menstrual period within the past 12 months, was not pregnant or lactating, and could read and understand English. Of the 820 women eligible, 620 agreed to participate (75.6 % of those eligible) and 508 actually began the study and provided initial cross–sectional data (81.9 % of those who initially agreed to participate) (See Table 2). 390 of the 508 women entered the longitudinal component of the study (76.8 % of the cross-sectional sample) by agreeing to provide data over time. A description of the characteristics of the women who agreed to participate in the longitudinal component (*N =* 390) and those who only completed the initial cross sectional component (*N =* 118) is shown in Table 3. Those who entered the longitudinal component compared to those who did not enter were more likely to be partnered, not a parent and not Black. There were no significant differences for education, employment, age, BMI, income and stress level.

**Table 2:** Smwhs sample identification and screening

Sampling Identification	N and (% of total enumerated)
Households enumerated	13,120 (100 %)
Households contacted by phone	11,222 (86 %)
Women in households 35–55 years of age screened	1,428 (11 %)
Women eligible after screening	820 (6 %)
Women who agreed to participate	620 (5 %)
Women who actually began study	508 (4 %)

**Table 3:** Baseline Sample Characteristics for women who participated in the Longitudinal Component Compared with women who participated only in the Cross Sectional Component (1990–1991)

	Women in Longitudinal Component (*n =* 390)	Women in Cross Sectional Component (*n =* 118)	
Characteristic	Mean (SD)	Mean (SD)	*p* value*
Age (years)	41.5 (4.3)	41.4 (4.4)	0.43
Years of education	15.7 (2.8)	15.3 (3.2)	0.15
Family gross income ($)	37360 (15,800)	35,500 (17,460)	0.27
Number live births	1.97 (1.4)	1.57 (1.4)	.006
Perceived stress	2.2 (0.55)	2.3 (0.55)	0.31
Characteristic	N (Percent)	N (Percent)	*p* value**
Currently employed			
Yes	336 (86.1)	102 (86.4)	0.94
No	54 (13.8)	16 (13.6)	
Race/ethnicity			
African American	32 (8.2)	26 (22.0)	.001
Asian /Pacific Islander	34 (8.7)	9 (7.6)	
Caucasian	311 (79.7)	80 (67.8)	
Other (Hispanic, Mixed)	13 (3.3)	3 (2.5)	
Marital Status			
Married/partnered	277 (71.0)	71 (60.2)	0.03
Never partnered/ divorced/widowed	113 (29.0)	47 (39.8)	
Never married/partnered	21 (7.2)	14 (6.5)	

*Independent *t*-test

**Chi-square test

**Fig. 2:**
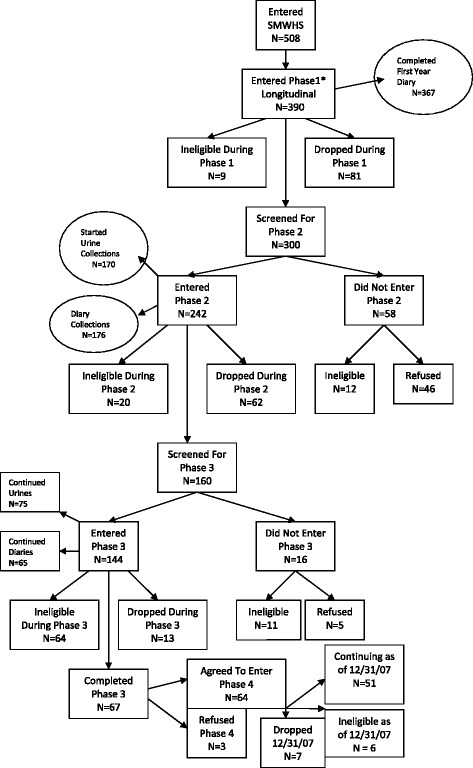
Retention Flow Chart. *Funding phases

For entry into the second major funding phase of the study (mid-1996), women still enrolled at the end of the previous phase of this longitudinal project, plus those who had dropped out of phase 1 but had contributed at least two years of data, were contacted by phone about participating in this second phase. A total of 300 women were contacted in mid-1996 and screened for continuing eligibility (5 years or less PM or, if taking hormones, age less than 60 years old, uterus intact and at least one ovary intact). Of those 300 women screened, 243 were eligible and agreed to enroll in phase 2 (62 % of the 390 who began the longitudinal component). In addition, between 2000 and 2002, 174 women provided a buccal cell smear for genotyping. See Fig. 2 for retention across the entire project.

For entry into the third major funding phase of the study (2001–2006) all eligible women (5 years or less PM or, if taking hormones, age less than 60, uterus intact and at least one ovary) who were still participating (*N =* 160) were contacted and screened (66 % of those who entered phase 2). Of these 160 women, 144 (90 %) agreed to continue for a third phase. At the end of phase three 67 women were still eligible and participating.

Research funds from the UW School of Nursing Research Intramural Funding Program were obtained in 2007 to continue data collection from those still eligible for the study. Of these 67 women, 64 were eligible and agreed to continue participation in the fourth and final phase until no longer eligible. This part of the study continued until February 2013 when all data collection was completed. Of the original 508 women who entered the study, by the end of the study in 2013, 173 had dropped due to personal reasons (34 %), 162 were lost to contact (32 %) and 173 became ineligible sometime during the study (34 %).

### Retention efforts

Numerous efforts were taken to retain the eligible sample throughout the study. These include the following:

- yearly birthday card with a personal note
- yearly thank you checks through the first two funding periods
- personal and consistent contact by the research staff
  - reminder postcards about data collection
  - in-person pick-up of urine and diaries at a community site or at home
  - reminder phone calls about pick-up of data
- flexibility regarding schedules; negotiating alternatives
- periodic sharing of findings with women
- yearly newsletter, The Midlife Times
- two Health Fairs at community sites
- a web site
- a certification of appreciation after 10 years of participation
- easy access to research staff via phone and email

### Data collection

In the first phase of the study all measures were pencil and paper measures. This included measures of symptom severity, stress, personal and social resources, socialization for midlife and aging, reproductive health experiences including menstrual cycle changes, social environmental demands, and personal health practices. These measures were obtained in an annual daily health diary across two to three menstrual cycles, an annual health questionnaire and a menstrual calendar.

In the second phase of the study measures of pituitary-ovarian and pituitary-adrenal function were added. These additional measures were obtained by collecting monthly first AM urine specimens on day 6 of the menstrual cycle, if the woman was still cycling. Women were instructed not to eat, drink, smoke, take medications or exercise before each urine collection. The health diary was collected on 3 consecutive days (days 5, 6 and 7) to coordinate with the time of the urine collection (day 6). For those with very erratic bleeding and those no longer having periods a consistent 3 days of the month was used for data collection. This procedure was used from late 1996 through 2000.

The data collection time for the diary and urine specimens was modified from 2001 through 2005. The timing was changed from monthly to quarterly for both the diary and urine collections. During all phases of the study the yearly health questionnaire and menstrual calendars were continued (See Table 4 for sample size for each measure by year).

**Table 4:** Frequencies for data sources (1990–2013)

	Questionnaire N	Diary N	Assay N (# specimens)
1990–1992	508	367	NA
1993	347	259	NA
1994	309	261	NA
1995	250	141	NA
1996	192	146	NA
1997	233	176	170 (1783)
1998	205	162	167 (1820)
1999	212	149	157 (1478)
2000	190	103	106 (1036)
2001	175	79	85 (340)
2002	157	65	74 (279)
2003	140	59	59 (236)
2004	110	46	54 (208)
2005	95	44	49 (179)
2006	84	30	NA
2007	57	20	NA
2008	47	18	NA
2009	37	15	NA
2010	31	10	NA
2011	20	10	NA
2012	17	5	NA
2013	12	5	NA

In addition, buccal cell smears were obtained from 174 of these women between 2000 and 2002. Urine collections stopped at the end of 2005. From 2006 to the end of the study quarterly health diaries, yearly health questionnaires and menstrual calendars, if still bleeding, were obtained.

### Data handling

Except for the interview at the start of the study, all data in phase 1 and phase 4 were collected by mail (yearly questionnaire, diaries, menstrual calendars). In phases 2 and 3 the diaries and urine samples were collected in person while the annual questionnaire and menstrual calendar were collected by mail. For the urine samples, after the first morning urine was collected by the participant it was immediately frozen in a home freezer at 0°. These specimens were either brought frozen to a community site by the participant at a prearranged time or were picked up by a research associate within 56 days (8 weeks) of collection. Each specimen was kept frozen during transport and then taken to the University of Washington School of Nursing Biobehavioral Lab and placed in a −70° centigrade freezer. The specimens were then assayed by the laboratory staff. (See Additional file 1: Assay Descriptions and Laboratory Assay Procedure). A maximum of 56 days for home freezing was determined by the laboratory staff using various intervals and testing for sample degradation. The diaries were picked up in a similar manner as the urine during phases 2 and 3. If urine was not collected, the diaries were mailed to the study personnel.

### Measures

A blank **menstrual calendar** was mailed at the end of each calendar year for completion during the following year. Any occurrence of bleeding (B) or spotting (S) was recorded. Beginning in 1996, the amount of B on a scale of 1 (light flow) to 4 (very heavy/flooding) was recorded with each occurrence. Spotting was any bloody vaginal discharge that did not require any protection [9]. (See Additional file 1 for sample calendar). The menstrual calendars were returned at the start of the following year and reviewed for completeness.

Definitions of bleeding events used for the study, called standard bleeding events, were modifications of those recommended by WHO [9] [Gray, RH. WHO Meeting on the Analysis of Bleeding Patterns, Feb 28, 1978, unpublished]. A standard bleeding episode was defined as ≥2 days of B or a mix of ≥2 B and S days but not all S days with ≤2 bleed free days. A standard bleeding interval was any series of ≥4 consecutive bleed-free days bounded by bleeding episodes. A bleeding segment was a bleeding episode and the subsequent bleeding interval.

The WHO standard definitions did not differentiate bleeding episodes from intermenstrual bleeding (IMB) or non-menses bleeding such as S or B days between consecutive bleeding episodes and within a bleeding interval. A limitation of the WHO standard definition of a bleeding episode (≥1 days B or S) was the creation of many very short bleeding segments. Short bleeding segments can overstate the incidence of irregularity, bias downward the age of onset of each MT stage and bias upward the duration of MT stages. To address this problem of short bleeding segments additional criteria were developed by the study staff and Sybil Crawford, PhD, to determine if a bleeding event with 1 B day or 1 or more S days only was an episode or IMB and whether 3 bleed free days between B or S days represented a bleeding interval or was part of the episode. The criteria were applied using the woman as the unit of analysis as recommended by Treloar [10] (See Additional file 1 for Nonstandard Bleeding Criteria). The basic premise behind these additional criteria was that the typical bleeding pattern of some women can reflect a slight variation from the standard definitions and that IMB or nonmenses bleeding is a phenomenon that needs to be accounted for as part of a woman’s bleeding pattern.

A reduction in the number of short bleeding segments was the result of this procedure. In the SMWHS sample. The majority of instances of 1 S day or ≥2 S days together occurred between episodes, in the bleeding interval (unpublished data).

After all the bleeding criteria were applied to the calendar data each calendar was assigned a subgroup for staging using staging criteria developed by the study personnel [11] and modified based on the findings of the ReSTAGE Collaboration [12] (See Additional file 1 for Staging Criteria).

A **health questionnaire** was mailed at the end of each year. This questionnaire obtained data about changes in health, the menstrual cycle, current health practices, medication use, stress, social support, mental health, symptoms and well-being. (See Additional file 1 for a summary of measures included in the annual health questionnaires).

A **health diary** was kept by a subset of the original 508 women. Initially this diary was kept daily for two to three menstrual cycles. It was completed once a year for three years (at the start of the study, 12 months later, and 24 months from the start). The data from this early diary was hand entered into the computer. In 1994 the diary was converted to a scannable format and for 1995 and 1996 was kept daily for two weeks once a year (around the time of the yearly health questionnaire). Beginning in late 1996 to the end of 2000 this scannable diary was kept for 3 days every menstrual cycle on days 5, 6, and 7, if there were identifiable menstrual periods, to correspond with the urine collection on day 6. Otherwise it was kept monthly on the same 3 days every month. Starting in 2001 the diary was completed once a quarter for the same 3 consecutive days instead of monthly. The diary included items such as symptoms commonly experienced by midlife women, medication use, stress levels and health practices (smoking, drinking alcohol, caffeine use, exercise, sleep). (See Additional file 1 for sample pages of the diary).

**Urine specimens** were obtained from a subset of women one time per menstrual cycle on day 6 or once a month if there were no identifiable periods. These urine collections began in late 1996 and continued until the end of 2005. This was a first morning specimen and was assayed for estrone glucuronide, FSH, total testosterone, cortisol, epinephrine and norepinephrine. (See Additional file 1 for assay descriptions).

A **buccal cell smear** was obtained for genetic analysis from 174 women sometime between 2000 and 2002. (See Additional file 1 for buccal cell smear collection procedure and Additional file 1 for genotyping sequencing).

### Analytic strategies

A variety of analytic strategies was used over the course of the study. Examples include discriminant function analysis [9], confirmatory factor analysis and LISREL [8–14], content analysis with cross tabulations [15–17], ANOVA and regression analysis [18], cluster analysis [19], t-tests [20], time series analysis [21], general estimating equation [22] and numerous papers since 2006 using multilevel modeling (MLM) [23–35]. The analytic method called multi-level modeling (MLM) was used for most of the longitudinal analyses once most of the data were collected and processed (from 2006 on). For all MLM analyses age was used as the measure of time. This method was specifically adapted for the SMWHS data by a statistician (Don Percival, PhD) and was developed using an R program to account for specific characteristics of the data such as an unbalanced design, serial correlation, and missing data [30]. (See Additional file 1 for a detailed description of the MLM procedure).

## Results

Selected results are presented to illustrate the contributions of each phase of the SMWHS. A complete list of publications from the Seattle Midlife Women’s Health Study is appended to the References section.

### Phase 1

Data collected during phase I of the study were used to amplify our understanding of women’s views of midlife and menopause, as well as to evaluate models of women’s health and health-seeking behavior during midlife. In response to open-ended questions, women described midlife as a time of many transitions: getting older and changing bodies, outlooks and relationships. Personal achievements and employment were central to the lives of midlife women in this study [16]. Women viewed menopause as a period of transition. When women were asked about their anticipation of menopause they indicated it was a time of uncertainty that elicited mixed feelings [17]. Women also revealed their meanings of menopause as the cessation of periods, experiencing the end of fertility and reproductive capacity, hormonal changes, new or different life stage, changing emotions, changing bodies, symptoms, and part of the aging process. Few referred to menopause as a time of risk for disease or of need for health care.

A model of depressed mood symptoms was developed, evaluating 3 pathways to depressed mood, comparing the influence of the MT, stressful life context, and health status pathways in a multiethnic sample (*N =* 337). The stressful life context pathway was most influential in accounting for depressed mood. Health status had a direct effect on depressed mood and an indirect effect through perceived stress. The menopausal changes pathway had little explanatory power. At the time this model was tested, the majority of participants were in the Late Reproductive stage or the Early MT stage. Nonetheless, these results suggested the need for clinicians to look beyond menopausal status to the broader context of midlife women’s lives [8].

The primary endpoint throughout the study was type and severity of symptoms women experienced and reported during the MT and early PM. When the symptoms women experienced during midlife were first examined, measured during the premenses week, several groups were identified, including: dysphoric mood, vasomotor, somatic, neuromuscular,and insomnia symptoms. Notably the stability of vasomotor and somatic symptoms was lowest over the three year period studied, but dysphoric mood, neuromuscular, and insomnia symptoms were relatively stable, suggesting their chronic experience in this cohort [13]. The variability of the vasomotor and somatic symptoms over the three year period led to a focus on the role of the MT and related hormonal changes during subsequent phases of the study.

During phase 1 women’s health-seeking behavior was also investigated and was then tracked during subsequent phases. After publication of Women’s Health Initiative findings in 2002 linking hormone therapy (HT) with increased risk of breast cancer, stroke, heart attacks and other health problems, the percent of women taking hormones during the MT decreased from 49 % in 1999 to 35 % in 2003 [23].

### Phases 2, 3, and 4

#### Development of a staging system

Phase 2 of the study focused on the development of a staging system for the MT that eventually informed and was integrated with the Staging Reproductive Aging Workshop (STRAW) efforts [36], and later validated by the multi-country work of the Re-STAGE Collaboration [11, 37–39]. Mitchell led development of the MT staging system from detailed observation and analysis of menstrual calendar data over a seven year period (1990–1997) [11]. Development of the staging system for the MT provided a useful framework to organize subsequent analyses and demonstrate the influence of the MT stages on endocrine patterns, symptoms, and other aspects of the MT.

An important measurement issue related to staging reproductive aging was whether retrospective and prospective reporting of menstrual irregularity by women would influence staging efforts. Agreement between women’s reporting on a menstrual calendar and questionnaires with retrospective reports was weak, thus we incorporated only prospective reporting on menstrual calendars in the SMWHS staging approach [40].

The original and modified stages and criteria for staging used by SMWHS were as follows:

##### Pretransition stage

when cycles were regular with no change in length of periods, amount of flow or cycle length from the previous year. This stage was later called Late Reproductive stage to correspond to STRAW recommendations.

##### Early stage

when cycles were still regular but there was a change in length of periods, amount of flow or cycle length from the previous year. This stage was later called Late Reproductive stage to correspond to STRAW recommendations.

##### Middle stage

when cycles became irregular, i.e., start of consecutive cycles were 7 or more days apart. This stage was later called Early stage to correspond to STRAW recommendations.

##### Late stage

when periods were skipped, i.e., twice the modal cycle length between consecutive cycles. The criteria for this stage were later changed to 60 or more days of amenorrhea between the start of consecutive periods to correspond to the findings from the ReSTAGE Collaboration [39].

The original focus of staging in the SMWHS was on the menopausal transition. When the Staging Reproductive Aging Workshop (STRAW) investigators proposed use of stages of reproductive aging across the lifespan, we adopted the STRAW staging approach derived from consensus of investigators who participated in the STRAW workship in 2001. Our initial staging system had included an early, middle, and late stage of the menopausal transition. Because the STRAW investigators believed that the menopausal transition did not begin until cycle intervals became irregular, we adapted our staging to fit their recommendations. We no longer used our old definition of early menopausal transition, which included regular cycles with more subtle changes in the length of the period and cycle length, and instead adopted the STRAW definition of early stage. We also changed our pretransition stage to use the nomenclature of STRAW: late reproductive stage.

**Age of onset** of MT stages and the final menstrual period (FMP), and **duration** of the Early and Late MT stages were identified. On average, women (*N =* 121) entered Early stage at age 46.4 (SD = 3.4) and stayed in the stage (*N =* 82) for an average of 2.8 years (SD = 1.5). On average, women (*N =* 130) entered Late stage at age 49.4 (SD = 2.7) and stayed in this stage (*N =* 84) for an average of 2.5 years (SD = 1.3). The average age (*N =* 114) for the FMP (start of PM) for this cohort was 52.1 (SD = 2.9) years [37].

To identify an onset of each MT stage it was necessary to have bleeding data about the prior stage for the previous 12 months so the time of change could be identified. For example, using the staging criteria, if a woman was in Early stage for one year and the next year met the criteria for Late stage, the onset of Late stage could be identified. However, if she was in Late stage for one year but the prior 12 months of calendar data were not available, her onset of Late stage would be unknown. This same situation also would apply to onset of Early stage. Content analysis of women’s descriptions of irregularity and skipping of periods revealed that using simple questions about these was not adequate to apply the staging criteria. Instead, it was important to use the menstrual calendars to collect actual bleeding data [40].

#### Hormonal changes across the menopausal transition

**Fig. 3:**
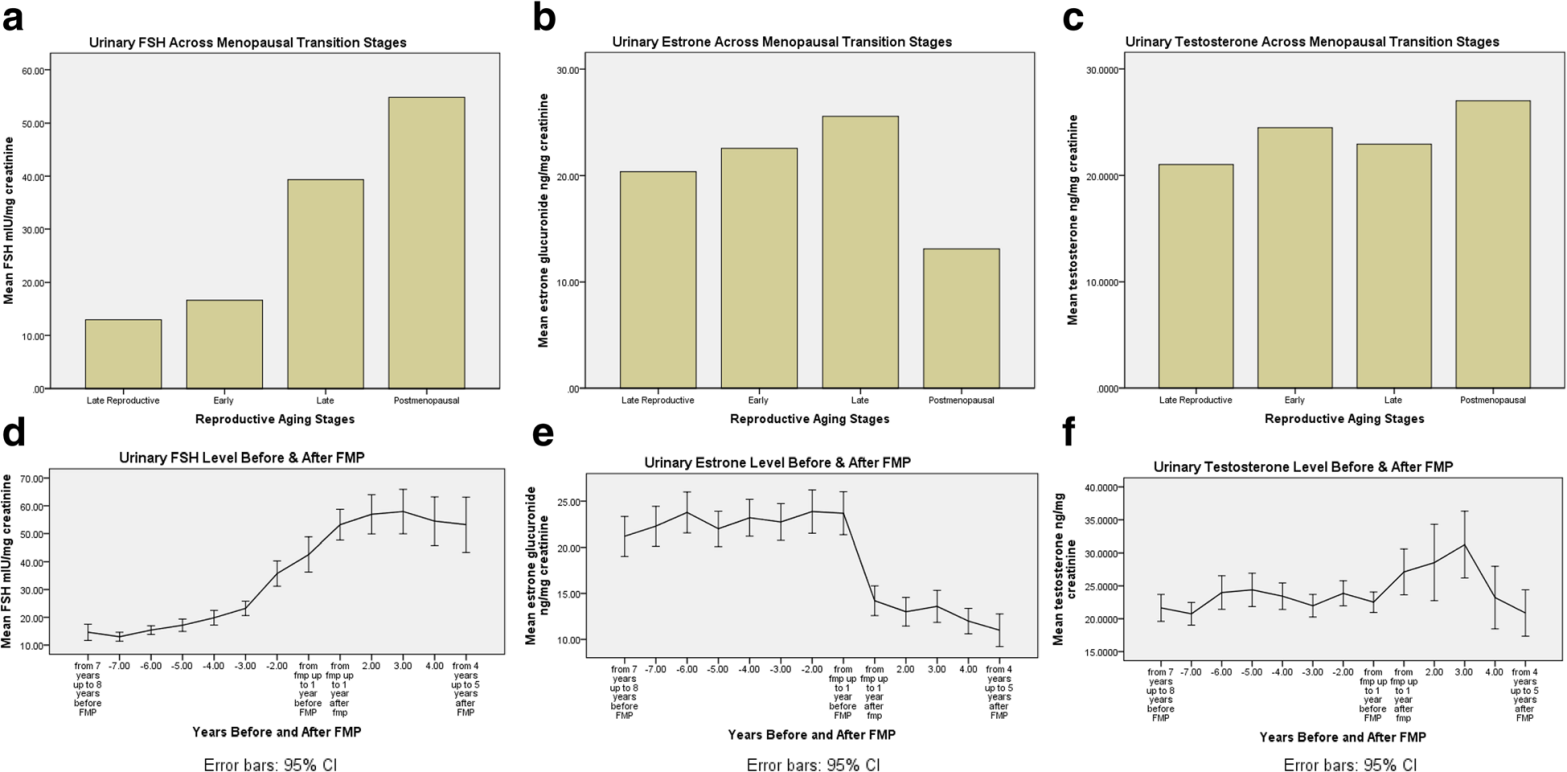
Endocrine Values Across Menopausal Transition Stages and Years Before and After the Final Menstrual Period (FMP). **a**. Urinary estrone across menopausal transition stages; 3**b**. Urinary Estrone Level before and after FMP; 3**c**. Urinary FSH across menopausal transition stages; 3**d**. Urinary FSH Level before and after FMP; 3**e**. Urinary Testosterone across menopausal transition stages; 3 **f**. Urinary Testosterone Level before and after FMP

An inspection of changes in urinary **FSH** (follicle-stimulating hormone) levels across the MT showed a rise as women progressed from Early MT to Late MT stage and to early PM and urinary e**strone** levels rose slightly from the Early to the Late MT stage and then dropped substantially the final year before and the first year after the FMP. **Urinary testosterone** levels remained flat across all MT stages and early PM. When these 3 hormones were analyzed for an association with MT stage across time, early PM had a significant negative effect on estrone and both Late MT stage and early PM had a significant positive effect on FSH. Testosterone was not affected by stage (unpublished data). (See Figs. 3 a,b,c)

When these same hormone levels were graphed based on number of **years before and after FMP** (from 8 years before to 5 years after FMP) FSH began to rise at 3 years before FMP and steadily increased to 3 years after FMP when it leveled off to at least 5 years FMP. Estrone showed a drop in level within 1 year before FMP and then slowly continued to decline to at least 5 years after FMP. Testosterone began to rise within 1 year before FMP, peaked at 3 year after FMP and declined steadily to at least 5 years after FMP (See Figs. 3 d,e,f).

Because of the important relationship of stress during midlife to symptoms, urinary **cortisol** was studied. The findings showed an increase in cortisol in the 7 to 12 months after onset of Late stage compared to the 7 to 12 months before onset of Late stage [20]. Also, women with increased cortisol levels during the Late stage had more severe hot flashes than those without a cortisol increase during the same stage [20]. In another study of cortisol using multilevel modeling there was a significant positive relationship between urinary epinephrine, norepinephrine, estrone, FSH, testosterone and hot flashes with cortisol levels in a univariate model. Health-related and social factors and symptoms other than hot flashes did not show a significant effect on cortisol levels. When the significant variables were combined in a multivariate model only estrone and FSH had a significant effect on cortisol [25].

**Fig. 4:**
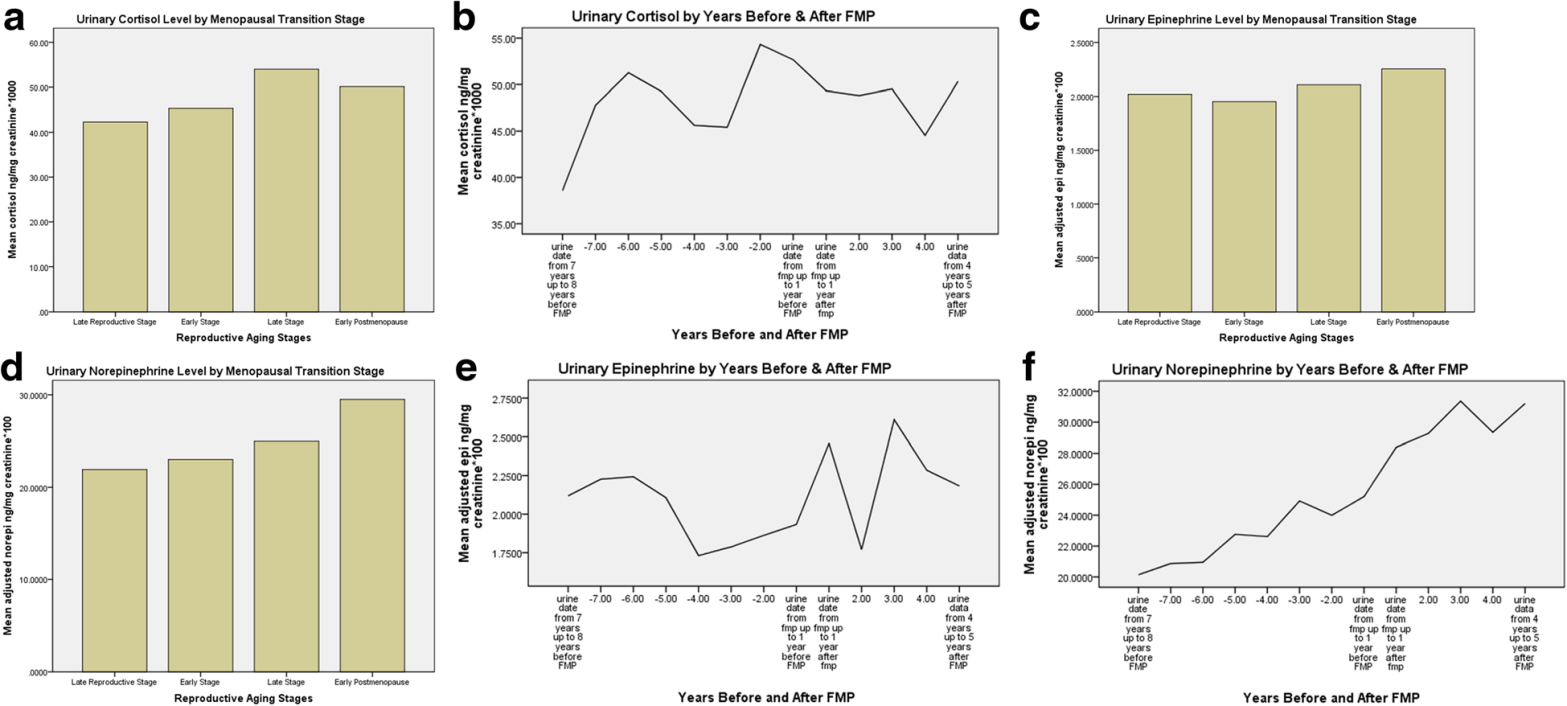
**a.** Urinary Cortisol by Menopausal Transition Stage. **b**. Urinary Cortisol by Years Before and After FMP. **c**. Urinary Epinephrine by Menopausal Transition Stages. **d**. Urinary Norepinephrine by Menopausal Transition Stages. **e**. Urinary Epinephrine by years Before and After FMP. f. Urinary Norepinephrine by Years Before and After FMP

An inspection of changes in urinary cortisol revealed a rise in the late MT stage, as seen in earlier analyses (See Fig. 4a) [15] and inspection revealed a gradual increase from 7 years before to 5 years after FMP (Fig. 4b). An inspection of urinary epinephrine and norepinephrine levels across MT stages showed a minimal change in epinephrine across stages and a slight rise in norepinephrine from Early MT stage to early PM (Fig. 4 c and d). When a multilevel analysis of these catecholamines across MT stage was done no significant effect of stage was found on epinephrine or on norepinephrine (unpublished data). In contrast, when number of years before and after FMP were examined, epinephrine showed no definitive pattern while norepinephrine slowly rose from 8 years before FMP to 5 years after FMP (Fig. 4 e and f).

#### Well-being and the menopausal transition

General well-being as measured by the 4 item subscale of the General Well-Being Scale [41] was positively associated with satisfaction with social support and a sense of mastery [27]. A decrease in well-being was associated with negative life events. Being in Late Stage of MT was associated with a decrease in well-being only in the univariate analysis.

#### Symptom patterns across the menopausal transition

Because the primary end points throughout the SMWHS were symptoms, of interest was identifying effects of MT stages on various types of symptoms. In addition, we used a general model (See Fig. 1) to guide analyses of women’s symptom experiences over time that included the following concepts and examples of indicators for each: menopausal transition factors, aging, health-related factors, stress-related factors, and other co-occurring symptoms. In the following paragraphs, findings related to each of the symptom groups studied are summarized.

#### Hot flashes

An analysis of women using and not using hormone therapy (HT) revealed that increases in hot flash severity were associated with late transition stage, early postmenopause, use of HT, duration of early transition stage, age of entry into early PM and level of FSH. Age of entry into early transition and estrone levels were associated with decreased hot flash severity. Not associated with hot flash severity were being in early transition stage, age of entry into or duration of late transition stage and all of the psychosocial (anxiety, stress, depressed mood) and lifestyle variables (BMI, activity level, sleep, alcohol use). Use of HT ameliorated but did not eliminate severe hot flashes [23].

Hot flash severity persisted through the MT stages, peaking in the Late MT stage and diminishing only after the second year PM. Hot flash severity was associated with being older, being in the Late MT stage or early PM, beginning the Late MT stage at a younger age and reporting greater anxiety. In a model including only endocrine factors, hot flash severity was significantly associated with higher FSH and lower estrone levels [34].

#### Sleep symptoms

Severity of **nighttime awakening** was significantly associated with age, Late MT stage and early PM, higher FSH, lower E1G, more severe hot flashes, depressed mood, anxiety, joint pain, backache, and perceived stress, history of sexual abuse, poorer perceived health, and less alcohol use [30]. Severity of **problems going to sleep** was associated with hot flashes, depressed mood, anxiety, joint pain, backache, perceived stress, history of sexual abuse, poorer perceived health, less alcohol use, and lower cortisol, but not with MT stages or hormone levels. Severity of **early morning awakening** was significantly associated with age, hot flashes, depressed mood anxiety, joint pain, backache, perceived stress, history of sexual abuse, poorer perceived health, but not MT stages, estrone, or FSH.

#### Depressed mood

Most women experienced the MT without a high level of depressed mood. A small group of women experienced worsening of their mood. Another small group experienced improvement in their mood [19]. Women with consistently depressed mood were more likely to have hot flashes, stress, history of premenstrual syndrome and postpartum blues than women with occasional depressed mood or those without depressed mood [19, 42].

Depressed mood symptoms (measured by CES-D scores) were associated with being in the Late MT stage, severity of hot flashes, life stress, family history of depression, history of postpartum blues, sexual abuse history, body mass index, and use of antidepressants. Hormonal levels and age of entry into and duration of Late MT stage were unrelated [24]. In another multivariate analysis, when covariates were examined individually, a decrease in depressed mood as a single symptom was associated with early PM, higher estrone, more exercise and being partnered. An increase in depressed mood was associated with perceived stress, a history of sexual abuse and more severe sleep disruption symptoms (problem getting to sleep, awakening at night, early morning awakening). FSH level, BMI, alcohol use, number of live births and hot flash severity were not associated with depressed mood. In a model with multiple covariates that individually had a significant effect, awakening at night no longer significantly increased depressed mood. Also, estrone level and early PM were no longer associated with a decrease in depressed mood [Mitchell, ES and Woods, NF Depressed Mood during the Menopausal Transition, Reproductive Aging and Life: Observations from the Seattle Midlife Women’s Health Study. Unpublished].

#### Cognitive symptoms

Women in the Late Reproductive and Early MT stages and those who used hormones reported more problems with memory measured by the Memory Functioning Questionnaire than women in Late stage [18]. About 72 % of women reported problems remembering names at least some of the time. About 50 % had a problem remembering where they put things, recent phone numbers, things others told them (or they told others), keeping up correspondence and forgetting what they were doing. However, none of these events was considered a serious problem [18]. Many types of problems with memory were related to lower ratings of health and depressed mood. Problems with current memory and remembering past events were associated with higher levels of reported stress, which women attributed to the burden of meeting multiple role demands [18].

**Memory changes** most noted by women (mean age 47 years) who responded to open-ended questions about their memory were difficulty remembering words or numbers, i.e., verbal memory. These changes were attributed to increased role burden and stress, getting older, physical health, menstrual cycle changes/hormones, inadequate concentration, and emotional factors [15].

As individual covariates and in a multivariate model, age, anxiety, depressed mood, night-time awakening, perceived stress, perceived health, and employment were each significantly related to **difficulty concentrating**. Hot flashes, amount of exercise and history of sexual abuse had a significant effect as individual covariates but not in the final multivariate model. The best predictors of **forgetfulness** when analyzed as individual covariates and in the multivariate model were age, hot flashes, anxiety, depressed mood, perceived stress, perceived health and history of sexual abuse [32].

#### Pain symptoms

Pain symptoms rose slightly with age. A significant increase in **back pain** was reported during the Early and Late MT stages and early PM, but urinary E1G, FSH and testosterone levels were unrelated. Of the stress-related factors, perceived stress and lower overnight urinary cortisol levels were associated with more severe back pain; history of sexual abuse and catecholamines did not have a significant effect. Women most troubled by symptoms of hot flashes, depressed mood, anxiety, night-time awakening, and difficulty concentrating reported significantly greater back pain. Of the health-related factors, having worse perceived health, exercising more, using analgesics, and having a higher body mass index were associated with more back pain, but alcohol use and smoking did not have significant effects. Of the social factors, only having more years of formal education was associated with less back pain; parenting, having a partner, and employment did not have significant. Factors associated with joint pain included age but not menopausal transition-related factors. Symptoms of hot flashes, night-time awakening, depressed mood, and difficulty concentrating were each significantly associated with **joint pain**. Poorer perceived health, more exercise, higher body mass index, and greater analgesic use were all associated positively with joint pain. History of sexual abuse was the only stress-related factor significantly related to joint pain severity [29].

#### Sexual desire symptoms

Women’s concerns about decreasing sexual desire during midlife prompted analysis of factors influencing sexual desire as recorded in the symptom diaries. Women reported a significant reduction in sexual desire during the Late MT stage and early PM. Those with higher urinary E1G and T reported significantly higher levels of sexual desire whereas those with higher FSH levels reported significantly lower sexual desire. Women using hormone therapy also reported higher sexual desire. Those reporting higher perceived stress reported lower sexual desire, but having a history of sexual abuse did not have a significant effect. Those most troubled by symptoms of hot flashes, fatigue, depressed mood, anxiety, difficulty getting to sleep, early morning awakening, and awakening during the night also reported significantly lower sexual desire, but there was no effect of vaginal dryness. Women with better perceived health and those reporting more exercise and more alcohol intake also reported greater sexual desire. Having a partner was associated with lower sexual desire [26].

#### Urinary incontinence symptoms

**Stress urinary incontinence** (SUI) was associated significantly with individual predictors of worse perceived health, history of ≥3 live births, being in the Early MT stage, having less formal education and being white. **Urge incontinence** (UUI) was associated significantly with individual predictors of increasing age, worse perceived health, BMI ≥30, history of ≥3 live births, and lower FSH levels. Both SUI and UUI were significantly associated with lower self-esteem and with age included in the models as a measure of time. UI effects on mood symptoms, attitudes toward aging and menopause, perceived health and consequences for daily life were not significant [22, 33].

#### Interference of symptoms with work and relationships

Women reported the effects of their symptoms on work and relationships in the symptom diary. Analyses of the extent to which symptoms interfered with daily living revealed that **interference with work** was significantly associated with perceived health, stress, hot flashes, depressed mood, anxiety, difficulty getting to sleep, awakening during the night, early morning awakening, backache, joint pain, forgetfulness and difficulty concentrating. **Interference with relationships** was significantly associated with age and individual covariates perceived health, estrone, perceived stress, depressed mood, anxiety, sleep symptoms, backache, joint pain, forgetfulness and difficulty concentrating [31].

#### Genetic influences and the menopausal transition

Polymorphisms in the estrogen synthesizing, metabolizing, and receptor genes were genotyped and associated with both symptoms and the timing of the events of the MT. Women with the CYP19 11r polymorphism reported more severe and frequent hot flashes during the Early and Late MT stages and early PM and higher E1G levels during Early and Late stages. [43]. In addition, polymorphisms in the 17 beta HSD gene (rs 5942 and rs 2389) were related to a symptom cluster incuding high severity hot flashes and moderate levels of 5 other symptom groups (sleep, mood, cognitive, pain symptoms). Moreover the rs2389 heterozygous allele had a significant positive effect on estrone and rs2830 homozygous mutant allele had a significant negative effect on FSH. The rs5942 17 HSD had no effect on either estrone or FSH (unpublished data).

Women with two CYP19 7r alleles had menarche earlier (11.5 y) than those with one CYP19 7r allele (13.1 y). Women with two CYP19 11r alleles were 2 years older at onset of Late stage than those with one CYP19 11r allele (50.7 y vs 48.6 y). Those with two CYP19 7r(−3) alleles were 2 years older at FMP than those without this allele (53.9 y vs 51.3 y). Women with the homozygous wild-type allele for HSDB1 (rs2830) were younger at FMP by 2 years than those with the heterozygous allele (50.8 y vs 52.9 y). Women with the heterozygous allele for CYP1B1*2 had a later age at menarche compared with women with the homozygous wild type (13 y vs 12.5 y). [44].

#### Stress and symptoms during the menopausal transition

Although some would contend that the MT is inherently stressful, factors that influenced the level of perceived stress among SMWHS participants were inadequate income to meet needs, lower levels of perceived health status, role burden and current employment [28]. Of interest was that perceived stress was related to each of the symptoms studied: hot flashes, depressed mood, lower sexual desire, difficulty getting to sleep, night-time awakening, early morning awakening, forgetfulness, difficulty concentrating, but not urinary incontinence symptoms. Perceived stress was not related to MT stage nor to the endocrine assays measured, including E1G, FSH, cortisol, and the catecholamines.

#### Symptom clusters associated with the menopausal transition

Analyses of each of the symptoms studied indicated they were commonly associated with other symptoms, e.g. hot flashes with sleep problems, depressed mood, pain and cognitive symptoms. The realization that women experienced multiple, co-occurring symptoms (defined as symptom clusters) during the MT and early PM led to further study [45]. Three symptom clusters composed of hot flashes and five groups of symptoms that had been identified in prior factor analysis (depressed mood symptoms, sleep disruption symptoms, tension symptoms, cognitive symptoms, and pain symptoms) among this community-based cohort [46]. Cluster I was composed of low severity hot flashes with low severity sleep disruption symptoms, depressed mood symptoms, tension symptoms, cognitive symptoms and pain symptoms (75 %); Cluster II was high severity hot flashes with a moderate level of the 5 symptom clusters (12 %); and Cluster III was low severity hot flashes with moderate severity levels of the 5 symptom clusters (13 %). When each of the 3 clusters were compared with each other for estrone, FSH, testosterone, epinephrine and norepinephrine significant group differences were between Cluster I (low hot flash/low symptom clusters) and Cluster III (high hot flash/moderate symptom clusters), and between Cluster I and Cluster II (low hot flash/moderate symptom clusters). Cluster III had lower estrone, higher FSH, lower epinephrine and higher norepinephrine than Cluster I and Cluster II had lower epinephrine levels than Cluster I. Cortisol and testosterone had no significant group differences among the 3 clusters [47].

When perceived stress levels were compared among the 3 clusters, Clusters II and III had significantly higher levels than Cluster I (unpublished data). Finally, polymorphisms in estrogen synthesis, metabolism, and receptor genes were tested. Only the 17HSD polymorphisms (rs 5942 and rs 2389) significantly differentiated Cluster III from Cluster I. None of the polymorphisms differentiated Cluster II from I or Cluster II from III.

## Conclusions and Discussion

Contributions of the SMWHS included:

- Development of a system for staging reproductive aging with emphasis on the period from the Late Reproductive stage through the early PM and establishment of the validity of the staging system with the ReSTAGE Collaboration and contributions to the Staging Reproductive Aging Workshop and STRAW + 10 [48];
- Incorporation of the staging system into the study of endocrine changes during the MT stages and early PM, including demonstration of changes in estrone, FSH, testosterone, cortisol, epinephrine and norepinephrine by MT stages and PM;
- Integration of the staging system into models of symptoms including hot flashes, sleep disturbances, depressed mood, pain, cognitive symptoms, incontinence, and sexual desire;
- Confirmation of effects of the MT stages and early PM on the following symptoms: hot flashes, awakening during the night, back pain, and sexual desire, but not on depressed mood, cognitive symptoms, incontinence, or joint pain;
- Identification of functional effects of symptoms on interference with work and relationships, in particular, effects of depressed mood and difficulty concentrating on work and depressed mood, anxiety, difficulty concentrating, and awakening during the night on relationships;
- Demonstration of effects of gene polymorphisms CYP 19 11r, 17 beta HSD (rs 2389 and 5942) in estrogen synthesizing genes on hot flashes as well as CYP 19 7r, CYP 19 7r(−3), 17 beta HSD (rs 2830) and estrogen metabolizing gene CYP 1B1*2 on events related to menarche and the MT; and
- Identification of naturally occurring symptom clusters and their relationship to endocrine levels (estrone, FSH), perceived stress, epinephrine, norepinephrine levels, and 17 beta HSD genotypes.

Results of this study can be generalized to women experiencing the natural menopausal transition and early postmenopause and who were not using hormone therapy. Limitations of the SMWHS included a predominantly White and well-educated sample, despite efforts to include Asian American and African American women. Another limitation was the smaller sample size relative to larger studies, such as the Study of Women and Health Across the Nation (SWAN) The limitation of sample size was compensated in part by the more frequent occasions of measurement, with some measures obtained several times per year. In addition, SMWHS was a longitudinal population-based study that enabled analysis of patterns observed in symptoms over time, up to 23 years for some participants. Efforts to recruit and retain a multi-ethnic sample were effective initially, but with waning retention during the latter years of the study. In addition, the development and application of specific criteria for staging the MT and analyzing data to examine effects of MT stages supported our ability to distinguish between endocrine factors, stress, and symptoms that were influenced by MT stages versus those who were not [44].

Issues for further study suggested by SMWHS included the importance of studying clusters of symptoms vs single symptoms and the need for interventions targeting multiple symptoms. We have begun examination of non-pharmacologic therapies that may be effective for clusters of symptoms vs individual symptoms [49–52]. In the interim, this research is being incorporated in the clinical education of women’s health care providers [53].

## Additional file

Additional file 1: Supplemental Information regarding Seattle Midlife Women's Health Study. (DOCX 5717 kb)

## Abbreviations

BMI: Body mass index
CYP: Cytochrome P450
E1G: Estrone
FMP: Final menstrual period
FSH: Follicle-stimulating hormone
HSD: Hydroxy steroid dehydrogenase
HT: Hormone therapy
MLM: Multi-level modeling
MT: Menopausal transition
PM: Postmenopause
SMWHS: Seattle Midlife Women’s Health Study
STRAW: Staging Reproductive Aging Workshop
SUI: Stress urinary incontinence
