# Supplementary material for: The Seattle Midlife Women’s Health Study: a longitudinal prospective study of women during the menopausal transition and early postmenopause
Source: Womens Midlife Health. 2016 Nov 9;2:6. doi: 10.1186/s40695-016-0019-x (PMC6299967; doi:10.1186/s40695-016-0019-x)
Supplement: Supplementary file 1 — Supplemental Information regarding Seattle Midlife Women's Health Study. (DOCX 5717 kb) [file 40695_2016_19_MOESM1_ESM.docx]

**Appendices**.

Appendix A. Assay descriptions and lab procedures

Appendix B. Sample Menstrual Calendar

Appendix C. Nonstandard Bleeding Criteria

Appendix D. Staging Criteria

Appendix E. Summary of Health Questionnaire Measures

Appendix F. Sample diary

Appendix G. Buccal cell smear collection procedure

Appendix H. Genotyping Sequencing

Appendix I. MLM Procedure

**APPENDIX A**

**Measures for the Urinary Assay Procedure**

Urine samples were preserved with sodium ethylenediaminetetraacetic acid and sodium metabisulfite and frozen at 7708C. All specimens, standards and controls were tested in duplicate and those with a coefficient of variance above 15% were repeated. A BioRad Quantitative Urine control and a pooled in-house urine control were included in all assays, and a member of the standard curve was repeated after every ten unknowns to monitor assay performance. In general, all samples from a calendar year were assayed during the next calendar year and multiple samples from each participant were assayed in the same batch during each year. All endocrine concentrations were corrected for variations in urine concentration by expressing the hormone level as a ratio to the concentration in the same urine specimen.

**Assay Descriptions**

**Urinary E1G**. Urinary E1G was selected as a marker for estrogen because it is stable, can be reliably measured without special preparation, and is highly correlated with serum estradiol levels (1-3). Urinary E1G was measured by a competitive enzyme immunoassay that cross-reacts 83% with estradiol glucuronide, thus measuring both estradiol and estrone in glucuronide forms. Baker and colleagues (4) found that 17A-estradiol-3-glucuronide is excreted in urine across the menstrual cycle in the same pattern as E1G with a clear midcycle peak but at concentrations five times lower than E1G. The E1G assay developed by O’Connor and colleagues captures meaningful patterns of change with respect to reproductive aging as demonstrated in previous studies of the MT(5) The assay is described in full elsewhere (3-6). All E1G concentrations were adjusted for hydration status using creatinine and corrected for nonparallelism (standardized to a 1:5 dilution) using the methods outlined in O’Connor et al (3,6). Average E1G concentrations for menopausal and cycling women as measured in our laboratory were obtained using daily specimens from one full cycle from each of 30 normally cycling US women (N = 799 specimens) and 30 daily specimens from 30 postmenopausal US women (N = 892); these specimens were not from the current study. Corrected for dilution factor and nonparallelism, E1G concentrations range from a minimum of 4.688 ng/mL to a maximum of 284.441 ng/mL, with an average of 35.919 ng/mL in cycling women, and from 1.879 to 44.575 ng/mL, with an average of 8.292 ng/mL in postmenopausal women (unpublished data). The lower limit of detection of the assay was 3.1 nmol/L. Average recovery from a urine matrix of low, medium, and high E1G standard doses was 101% (3). Intra-assay and interassay CVs were 2.1% and 9.6%, respectively, for an external (Bio-Rad) urine control (mean concentration, 2.144 ng/mL); the intra-assay and interassay CVs for an internal urine control (mean concentration, 1.586 ng/mL) were 2.8% and 14.5%, respectively. There was no evidence of trending in the urine control specimens across the study: the interassay CV from 255 microtiter plates was 10.1% for the internal control and 10.8% for the external control. The E1G enzyme immunoassay has slight nonparallelism, which is corrected statistically (3). Measures of urinary E1G in our sample are expressed in nanogram per microgram of creatinine.

**Urinary FSH**. FSH was assayed using Siemens Double Antibody FSH Kit. FSH levels assayed in urine were parallel with serum profiles obtained from reproductive-aged women over the menstrual cycle (7). The FSH RIA was designed for the quantitative measurement of FSH in serum and urine. To test for urinary FSH, a 2-mL aliquot of each urine sample was pretreated with an extraction solvent (È1 M sodium acetate/acetic acid), followed by overnight incubation in cold acetone. The resulting precipitate was then resuspended in a solution of calibrator matrix before assay. To test for fidelity of recovery using the extraction protocols, several calibrator standards obtained from the kit were used, each containing a known amount of FSH in a matrix solution. Recoveries for extraction using this method were on the order of 95%. The protocol for urinary extraction of FSH described in the product insert was followed. In our laboratory, the reporting range for urine FSH was 2.0 to 100 mIU/mL, the minimal detectable concentration was 1.6 mIU/mL. The interassay variation (run to run) was 7.1%, and the intraassay variation (within run) was 3.7% (N = 205).

**Urinary Testosterone**. Levels were assayed using the Siemens Total Testosterone Kit, a solid-phase RIA using a T-specific antibody immobilized to the wall of a polypropylene tube. A hydrolysis step preceded the assay and was accomplished by addition of concentrated hydrochloric acid to the urine specimens. The acidified samples were then boiled for 15 minutes to remove any interfering substances from the urine. The hydrolyzed urine was diluted into a T-free zero matrix, incubated in the tubes, and then evaluated by RIA. Labeled 125I-testosterone was added to all samples and competed with the T present in the sample for binding sites on the tube walls during a 3-hour incubation at 37-C. The unbound T was decanted and the remaining 125I-testosterone determined by measuring counts per minute using a Cobra Series Auto-Gamma Counting System. The calibration curve was prepared using standards ranging from 0 to 400 ng/dL. To assess the validity of the hydrolysis extraction of urine T, several calibrator standards were obtained from the kit to calculate the efficiency of recovery. Standards ranging from 25 to 400 ng/dL were used. The average recovery was 2.7% and ranged from 86.1% to 106%. The interassay variation was 12.38% (N = 791), and the intraassay variation (within run) was 8.75%. T levels used in the analysis were reported as nanograms of T per milligram of creatinine.

**Urinary Cortisol**. Urine cortisol levels were determined by radioimmunoassay (RIA) using a Coat-A-Count Cortisol Kit (Siemens Medical Solutions, Los Angeles, CA). Coat-A-Count Cortisol was designed for the quantitative measurement of unbound cortisol (hydrocortisone, Compound F) in serum, urine, and heparinized plasma. The assay is highly specific for cortisol and has extremely low cross-reactivity with other steroids, except for prednisolone. All participants using prednisone or prednisolone were excluded from analyses. The protocol for extraction of cortisol from urine provided by Siemens was followed: urine samples were mixed with dichloromethane (DCM) and centrifuged to separate the two phases, and the aqueous phase was then removed by aspiration. One hundred microliters of organic phase containing cortisol was transferred into tubes coated with an anticortisol antibody. The tubes were evaporated to complete dryness using a gentle stream of nitrogen. Twenty five microliters of the Zero Calibrator (Siemens Medical Solutions) was introduced to the tubes to rehydrate the evaporated samples. One milliliter of 125IYlabeled cortisol was then introduced for 45-minute incubation at 37-C. The unbound 125IYlabeled cortisol was decanted, and the bound fraction was measured using a Cobra Series Auto-Gamma Counting System. Results were determined by interpolation from a logit-log representation of the calibration curve. The curve was established using six calibration standards ranging from 0 to 50 Kg of cortisol per deciliter. Nonspecific binding was subtracted from all tubes. Standards ranging from 5 to 50 Kg/dL were used throughout the study to calculate variances. In our laboratory, the reporting range for this urinary cortisol assay was 1 to 50 Kg/dL, and the detectable concentration was 0.2 Kg/dL. Interassay precision was calculated for each of the three samples from the results of 20 extractions each. The CV (interassay) ranged from 8.2% to 12.5% for samples ranging from 0.9 to 8.3 Kg/dL. The intra-assay CV was 4.6% (N = 376) using a pooled in-house control (3.6 Kg/dL). There were no significant differences in cortisol values when the DCM extraction was compared to values obtained, with an additional chromatographic purification step using a disposable SepPak C-18 cartridge, after initial DCM extraction. Recovery rates ranged from 88.4% to 96.5% when samples were spiked with three cortisol solutions of 5, 10, and 20 Kg/dL.

**Urinary Catecholamines.** Epinephrine and norepinephrine were assayed by high performance liquid chromatography after extraction on Bio-Rex cation exchange resin (Bio-Rad) followed by aluminum oxide (Bio-Rad) precipitation using a modification of the LCEC Application Note No. 15 (Bioanalytical Systems, 1982). Briefly, a 500-KL sample of urine is treated with phosphate buffer before loading onto the resin bed and is then neutralized with distilled water. After treatment with 0.7 M sulfuric acid, the catecholamines were extracted from the resin and onto a matrix of powdered alumina using a solution of 2 M ammonium sulfate. After a 10-minute mixing period, the products awee extracted with perchloric acid. This eluant was then filtered and then injected onto a Microsorb C-18 column (Rainin) using an autosampler and analyzed with a Coulochem II electrochemical detector (ESA, Inc). Data acquisition was handled by EZCHROM software (Scientific Software, Inc). An internal standard, 3,4-dihydroxybenzylamine, was mixed with all standards and unknowns before extraction. All data were quantitated on peak area ratios, using a standard curve generated in each batch run. The intra-assay variation was 4.7%, and the interassay variation was 7.85%.

References

1. Denari JH, Farinati Z, Casas PR, Oliva A. Determination of ovarian function using first morning urine steroid assays. *Obstet Gynecol* 1981;**58**:5-9
2. Stanczyk FZ, Miyakawa I, Goebelsmann U. Direct radioimmunoassay of urinary estrogen and pregnanediol glucuronides during the menstrual cycle. *Am J Obstet Gynecol* 1980;**137**:443-450.
3. O'Connor KA, Brindle E, Holman DJ, et al. Urinary estrone conjugate and pregnanediol 3-glucuronide enzyme immunoassays for population research. Clin Chem 2003;49:1139-48.
4. Baker, TE, Jennison, KIM, Kellie, AE. The direct radioimmunoassay of oestrogen glucuronides in human female urine. Biochem J 1979, 177(2),729-738.
5. Ferrell, RJ, O’Connor, KA, Holman, DJ, Brindle, E, Miller, RC, Schecthter, DE, Gorrindo, T, Korshalla, L, Simon, J, Voda, A, Wood, JW, Mansifled, PK, Weinstein, M (2005) Monitoring the Trasition to menopause in a five year prospective study: aggregate and individual changes in steroid hormones and menstrual cycle lengths with age. Menopasue 12(5):567-577.
6. O'Connor KA, Brindle E, Shofer JB, et al. Statistical correction for non-parallelism in a urinary enzyme immunoassay. *J Immunoassay Immunochem* 2004;**25**:259-278.
7. Qui, Q, Overstreet, JW, Todd, H, Nakajima, ST, Steward DR, and Lasley, BL. Total urinary follicle stimulating hormone as a biomarker for detection of early pregnancy and periimplantation spontaneous abortion. Environmental Health Perspectives 1997:105(8):862-866.

**APPENDIX B**

**Menstrual Calendar**

**Seattle Midlife Women's Health Study**

**University of Washington**

**School of Nursing**

**IDNO 123 Year 2005**

| **Day** | **1** | **2** |  | **3** | **4** | **5** | **6** | **7** | **8** | **9** | **10** | **11** | **12** | **13** | **14** | **15** | **16** | **17** | **18** | **19** | **20** | **21** | **22** | **23** | **24** | **25** | **26** | **27** | **28** | **29** | **30** | **31** |
| --- | --- | --- | --- | --- | --- | --- | --- | --- | --- | --- | --- | --- | --- | --- | --- | --- | --- | --- | --- | --- | --- | --- | --- | --- | --- | --- | --- | --- | --- | --- | --- | --- |
| **JAN** | **B** | **B** |  | **B** | **B** | **B** |  |  |  |  |  |  |  |  |  |  |  |  |  |  |  |  |  |  |  |  | **B** | **B** | **B** | **B** | **B** |  |
| **FEB** |  |  |  |  |  |  |  |  |  |  |  |  |  |  |  |  |  |  |  |  | **S** | **B** | **B** | **B** | **B** |  |  |  |  |  |  |  |
| **MAR** |  |  |  |  |  |  |  |  |  |  |  |  |  |  |  |  | **B** | **B** | **B** | **B** | **B** | **B** |  |  |  |  |  |  |  |  |  |  |
| **APR** |  |  |  |  |  |  |  |  |  |  |  | **S** | **B** | **B** | **B** | **B** | **B** | **B** | **B** | **B** | **B** | **B** | **B** | **S** |  |  |  |  |  |  |  |  |
| **MAY** |  |  |  |  |  |  |  |  |  |  |  |  | **B** | **B** | **B** | **B** | **B** | **S** |  |  |  |  |  |  |  |  |  |  |  |  |  |  |
| **JUN** |  |  |  |  |  |  |  |  | **S** | **B** | **B** | **B** | **B** | **B** | **B** | **B** | **B** | **B** | **B** | **S** |  |  |  |  |  |  |  |  |  |  |  |  |
| **JUL** |  |  |  |  |  |  |  |  |  |  |  | **B** | **B** | **B** | **B** | **B** | **B** | **B** | **B** | **B** | **S** |  |  |  |  |  |  | **S** |  |  |  |  |
| **AUG** |  |  |  |  |  |  |  |  |  | **S** | **S** | **S** |  |  |  |  |  |  |  |  |  |  |  |  |  |  |  |  |  |  |  |  |
| **SEP** |  |  |  |  |  |  |  |  |  |  |  |  |  |  | **S** | **B** | **B** | **B** | **B** | **B** | **S** |  |  |  |  |  |  |  |  |  |  |  |
| **OCT** |  | **B** |  | **B** | **B** | **B** | **B** | **S** |  |  |  |  |  |  |  |  |  |  |  |  |  |  | **S** | **B** | **B** | **B** | **B** | **B** | **B** | **B** | **S** | **S** |
| **NOV** |  |  |  |  |  |  |  |  |  |  |  |  |  |  |  |  |  |  |  |  |  |  |  |  |  | **B** | **B** | **B** | **B** | **B** | **B** |  |
| **DEC** | **B** | **S** |  |  |  |  |  |  |  |  |  |  |  |  |  |  |  |  |  |  |  |  | **S** | **B** | **B** | **B** | **B** | **B** | **S** |  |  |  |

**For every day you spot or bleed enter an S or B in the appropriate square.**

**Record a 1, 2, 3 or 4 next to every B day.**

**(1: light flow, 2: moderate, 3: heavy, 4: very heavy/flooding)**

**For any month no bleeding or spotting occurs write in NO BLEEDING.**

**If you forget to record for a month write in FORGOT TO RECORD.**

**APPENDIX C. NONSTANDARD BLEEDING CRITERIA**

**Purposes:**

1. to determine if 3 bleed free days bounded by at least 1 B or S day is a bleeding episode or a bleeding interval using a woman’s own bleeding pattern;
2. to differentiate a bleeding episode of 1 day or ≥1 spotting days from intermenstrual or nonmenses bleeding.

**Rationale for SMWHS Nonstandard Bleeding Criteria:**

The strict application of the standard bleeding criteria originally recommended by WHO can result in very short bleeding segments, with the potential effect of over-stating the incidence of irregularity, biasing downward the age of onset of each MT stage and biasing upward the duration of MT stages.

The basic premise behind the NBE is that the typical bleeding pattern of some women can reflect a slight variation from the standard definitions and that IMB is a phenomenon that needs to be accounted for as part of a woman’s bleeding pattern. None of the standard definitions of a bleeding episode or interval account for IMB and not all bleeding or spotting represents a bleeding episode.

**Definition of a Nonstandard Bleeding Event**

A Nonstandard Bleeding Event (NBE) is a bleeding event that does not fit the standard definition for a bleeding episode or interval and must be assessed using additional criteria and the individual woman as the unit of analysis. A reference period of 365 days is used for determining each woman’s usual bleeding pattern.

**Types of Nonstandard Bleeding Events**

1. 3 bleed-free days bounded by 1 or more days of bleeding (B) and/or spotting (S),

2. 2 or more spotting days bounded by 3 or more bleed-free days,

3. 1 bleeding day or 1 spotting day bounded by 3 or more bleed-free days.

**Examples of Nonstandard Bleeding Events Highlighted**

| Day | **1** | **2** | **3** | **4** | **5** | **6** | **7** | **8** | **9** | **10** | **11** | **12** | **13** | **14** | **15** | **16** | **17** | **18** | **19** | **20** | **21** | **22** | **23** | **24** | **25** | **26** | **27** | **28** | **29** | **30** | **31** |
| --- | --- | --- | --- | --- | --- | --- | --- | --- | --- | --- | --- | --- | --- | --- | --- | --- | --- | --- | --- | --- | --- | --- | --- | --- | --- | --- | --- | --- | --- | --- | --- |
| JAN |  |  |  |  |  |  |  | **S** | **S** | **B** | **B** | **B** | **B** | **S** | **S** |  |  |  |  |  |  |  |  |  |  |  |  |  |  |  |  |
| **FEB** |  |  |  | **S** | **S** | **B** | **B** | **B** | **S** |  |  |  |  |  |  |  | **S** | **S** |  |  |  |  |  |  |  |  |  |  |  |  |  |
| **MAR** | **S** | **S** | **B** | **B** | **B** | **S** | **S** |  |  |  |  |  |  |  |  |  |  |  |  |  |  |  |  |  |  |  | **S** | **S** | **B** | **B** | **B** |
| **APR** | **S** |  |  |  |  |  |  |  |  |  | **S** |  |  |  |  |  |  |  |  |  |  |  |  |  |  |  | **B** |  |  |  |  |
| **MAY** |  |  |  |  |  |  |  |  |  |  |  |  |  |  |  |  |  | **B** | **S** |  |  |  | **B** | **B** | **B** | **S** |  |  |  |  |  |
| **JUN** |  |  |  |  |  |  |  |  |  |  |  |  |  |  |  |  | **S** | **B** | **B** | **B** | **S** |  |  |  |  |  |  |  |  |  |  |
| **JUL** |  |  |  |  |  |  |  |  |  |  |  |  |  |  | **B** | **B** | **B** | **B** | **S** |  |  |  |  |  |  |  |  |  |  |  |  |
| **AUG** |  |  |  |  |  |  | **B** | **B** | **B** | **B** | **S** |  |  |  |  |  |  |  |  |  |  |  |  |  |  |  |  |  |  |  |  |
| **SEP** |  |  | **S** | **B** | **B** |  | **S** | **S** |  |  |  |  |  |  |  |  |  |  |  | **S** | **S** | **S** |  |  |  |  |  |  |  |  |  |
| **OCT** |  | **B** | **B** |  |  |  |  |  |  |  |  |  |  |  |  |  |  |  |  |  |  |  |  |  |  | **S** |  |  | **B** | **B** | **B** |
| **NOV** | **S** | **S** | **S** |  |  |  |  |  |  |  |  |  |  |  |  |  |  |  |  |  |  |  |  | **B** | **B** | **B** | **S** | **S** | **S** |  |  |
| **DEC** |  |  |  |  |  |  |  |  |  |  |  |  |  |  |  |  |  | **S** | **B** | **B** | **B** | **S** | **S** |  |  |  |  |  |  |  |  |

**Bleeding events not highlighted are Standard, i.e., meet the standard definition**

**General Procedure for Applying Nonstandard Bleeding Criteria**

A reference interval is first identified in order to use the woman’s own data when assessing whether a bleeding event outside the standard definition is an episode or is IMB.

The coding is done to be as consistent as possible with other bleeding events within the reference interval. The preferred reference interval is 365 days before the event to be coded.

The coding procedure includes determination of the pattern of regularity within the reference interval, identification of a minimum flow score and the median episode length. Details for applying these criteria can be obtained from the Investigator (ESM) at nellem@uw.edu.

**APPENDIX D**

**MENOPAUSAL TRANSITION STAGING CRITERIA**

Using menstrual calendar data, women not taking any type of estrogen or progestin were classified according to stages of reproductive aging: Late Reproductive, Early MT, Late MT, or early postmenopause, based on staging criteria developed by Mitchell, Woods and Mariella, 2000 and validated by the ReSTAGE collaboration (Harlow, 2006, 2007, 2008). The names of stages match those recommended at the Stages of Reproductive Aging Workshop STRAW) (Soules, 2001). The time before the onset of persistent menstrual irregularity during mid-life was labeled the Late Reproductive stage when cycles were regular. Early stage was defined as persistent irregularity of more than 6 days absolute difference between any two consecutive menstrual cycles during the calendar year, with no skipped periods. Late transition stage was defined as persistent skipping of one or more menstrual periods.

.A skipped period was defined as 60 or more consecutive days of amenorrhea during the calendar year. Persistence meant that the event, irregular cycle or skipped period, occurred one or more times in the subsequent 12 months. Final menstrual period (FMP) was identified retrospectively after 1 year of amenorrhea without any known explanation. The date of the FMP was considered synonymous with the term menopause. Early postmenopause was within 5 years of the FMP.

**APPENDIX E**

Table of Concepts, Variables, and Occasions of Measurement**
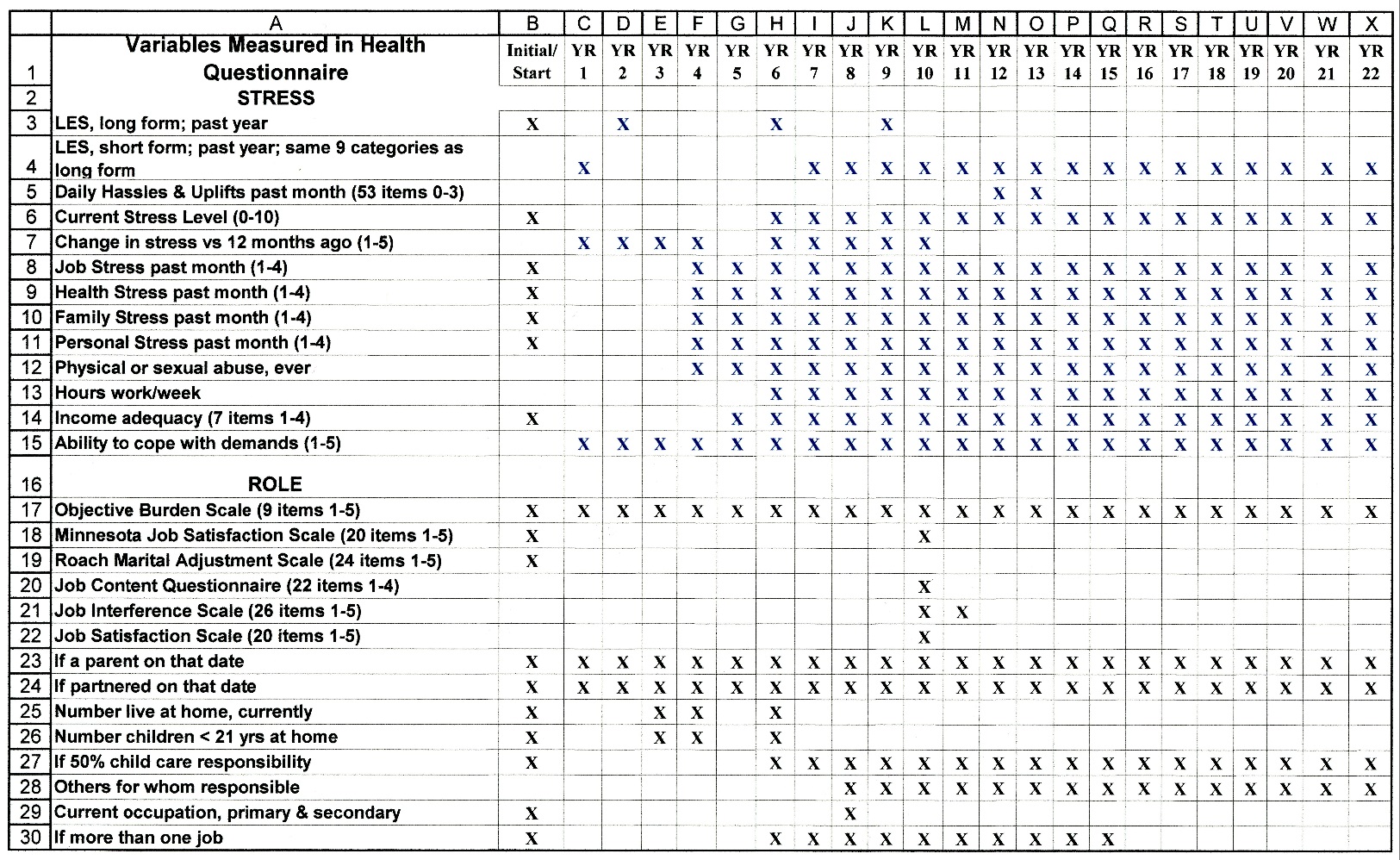

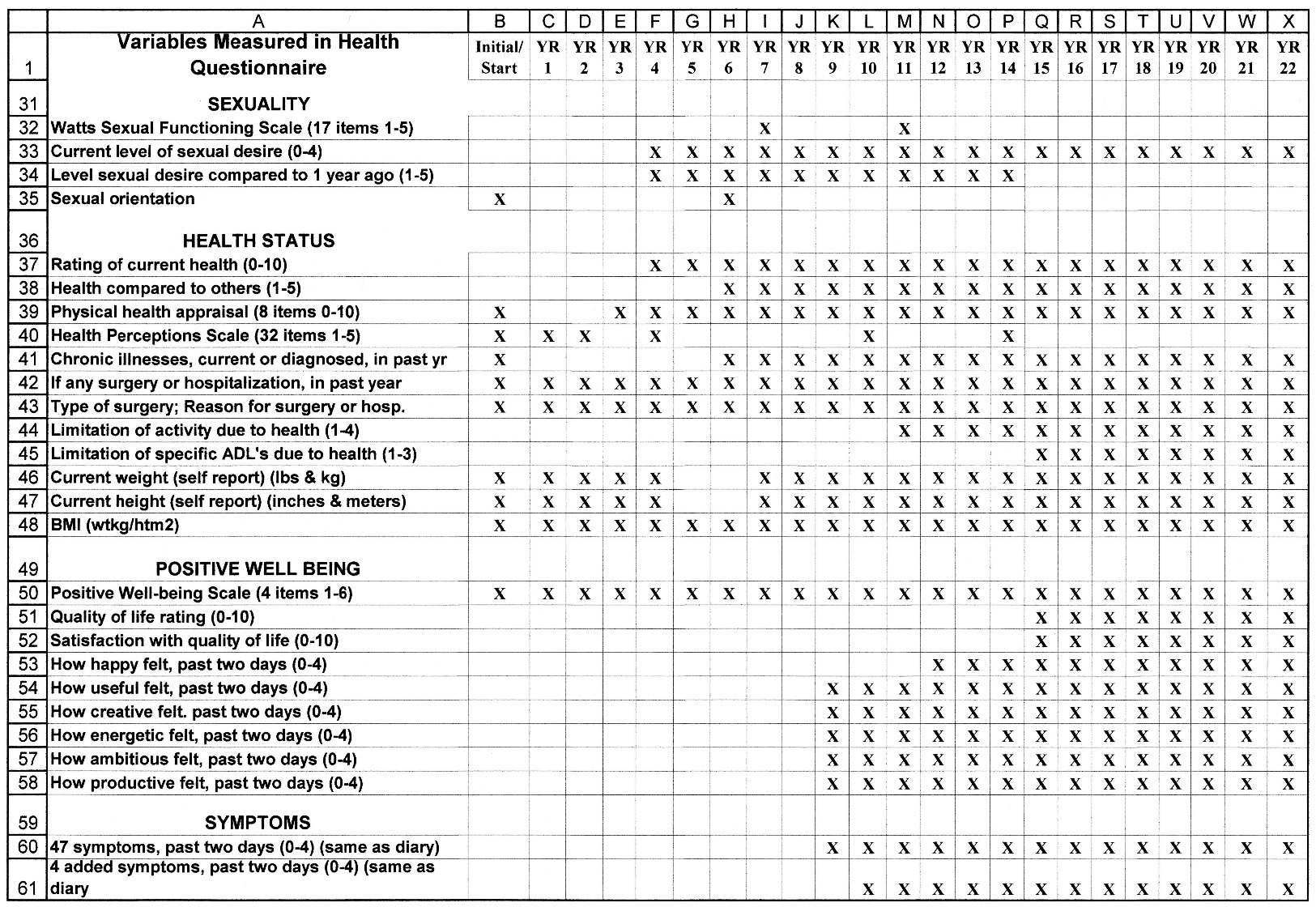
**

**
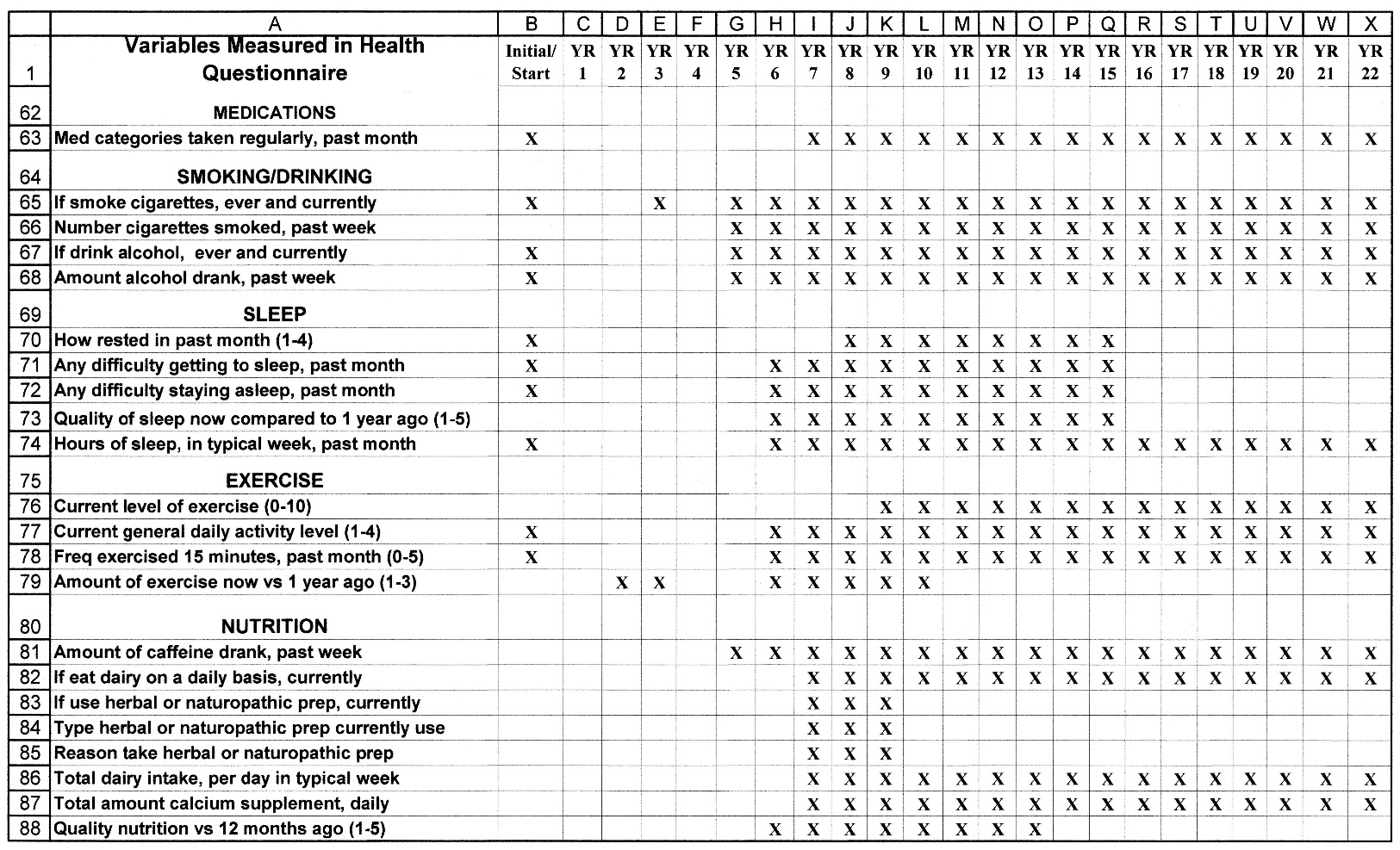
**

**
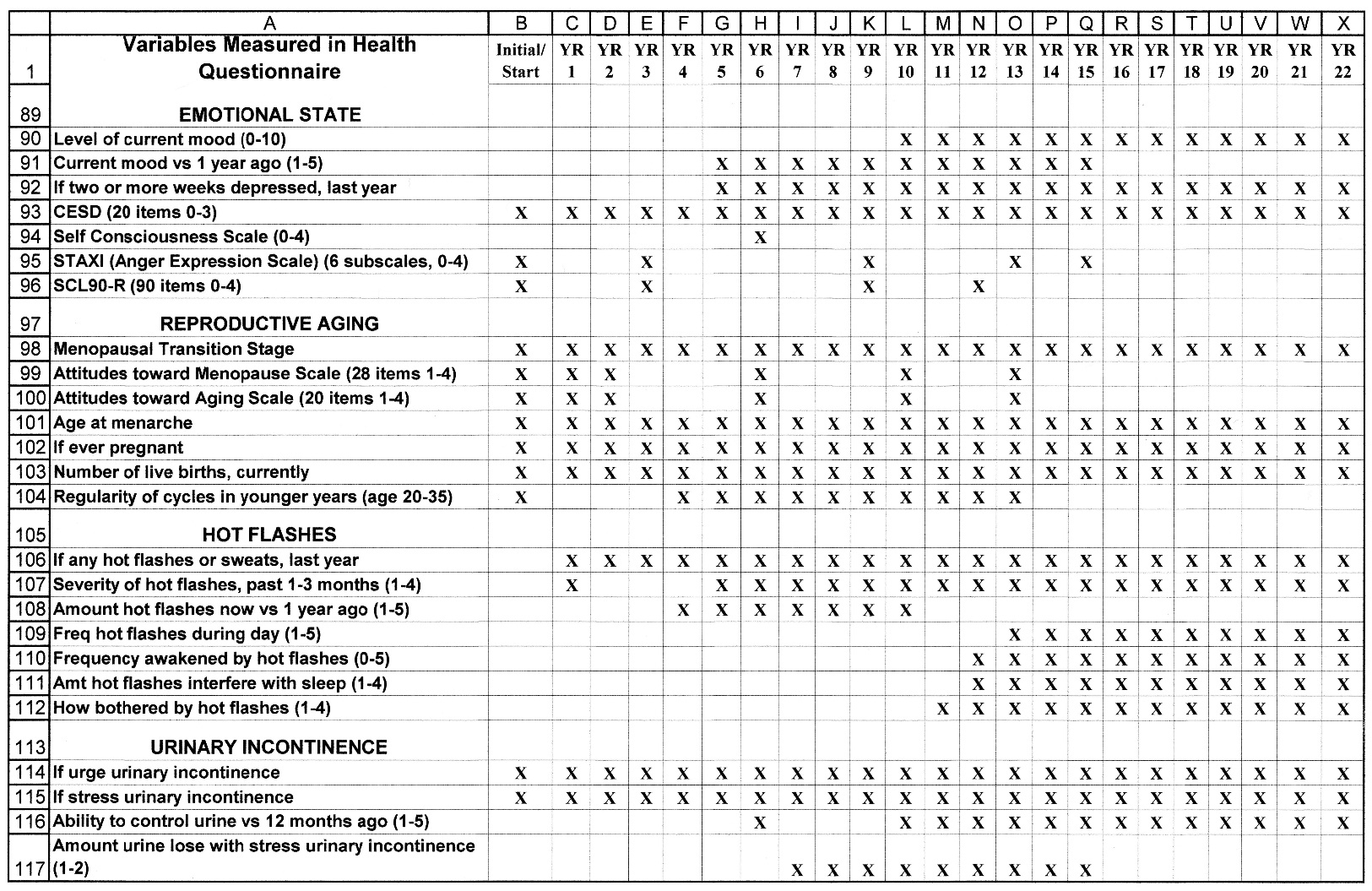
**

**
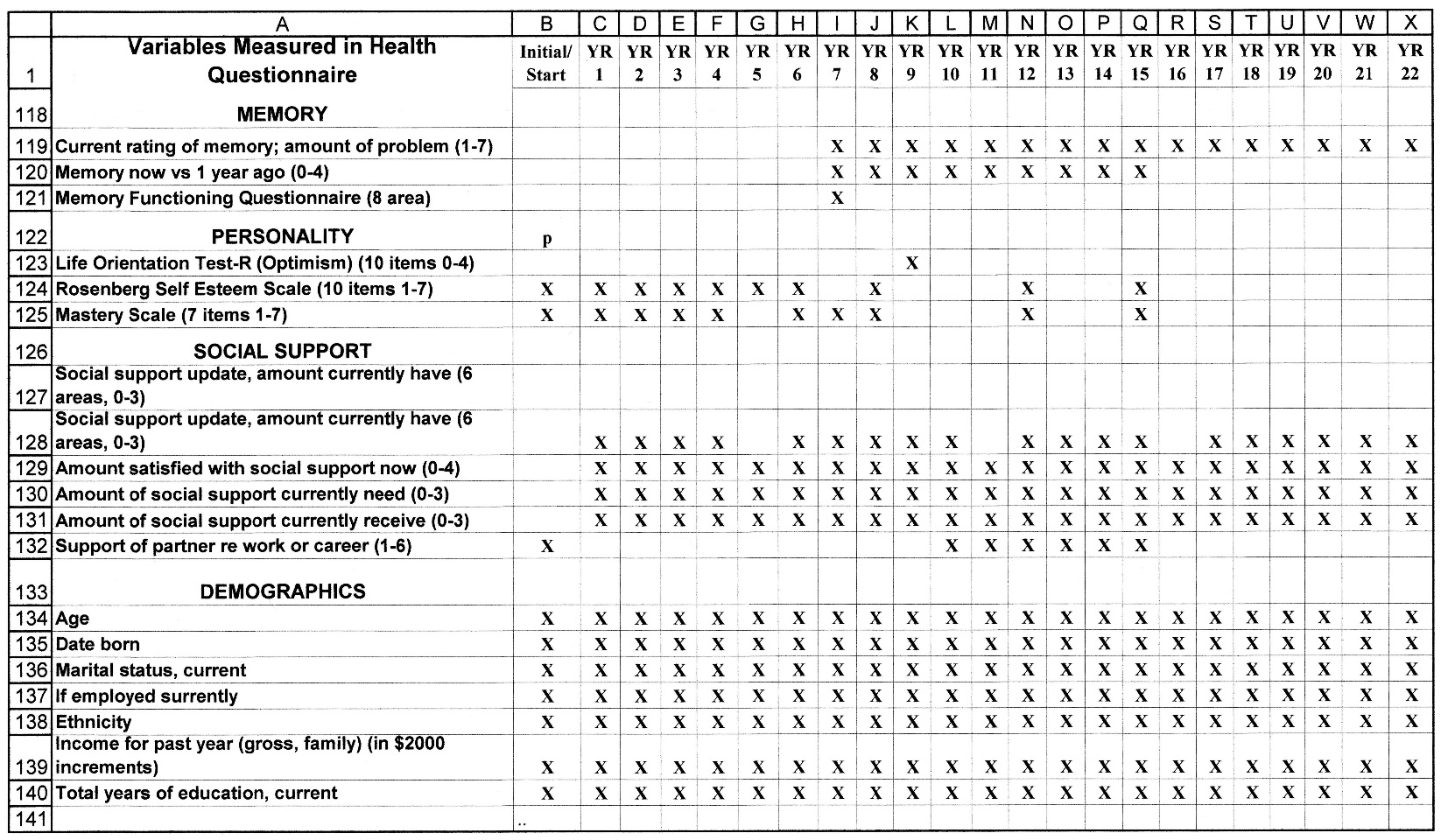
**

**References for Standardized Instruments**

**Attitudes toward Menopause**

Neugarten B, Wood V, Kraines R, Loomis B. Women's attitudes toward the menopause. Vita Humana 1963; 6:140-151.

**CESD – Center for Epidemiologic Studies Depression Scale**

Radloff L. The CES-D scale: A self report depression scale for research in the general population. Applied Psychological Measurement 1977; 1(3):358-401.

**Health Perceptions Scale**

Ware, J. E. (1976). Scales for measuring general health perceptions. *Health Services Research, 11*(4), 396-415.

**Positive Well-being Scale**

Brook, R.H., Ware, J.E., Jr., Davies-Avery, A., Stewart, A.L., Donald, C.A., Rogers, W.H., et al. (1979). Overview of adult health measures fielded in Rand's health insurance study, ch 6—findings and conclusions. *Medical Care*, 1979, *17*:16–55.

**Optimism – Life Orientation Test**

Scheier, MF, Carver, CS & Bridges, MW. Distinguishing optimism from neuroticism (and Trait Anxiety, Self-Mastery, and Self-Esteem): A reevaluation of the Life orientation Test. J of Personality and Social Psychology 1994,, 67:1063-=1078.

**Self Consciousness**

Fenigstein, Allan, Scheier, Michael F. & Buss, Arnold H. Public and private self-consciousness: assessment and theory. Journal of Consulting and Clinical Psychology 1975, 43(4), 522-527.

**Mastery**

Pearlin LI, Schooler C. The structure of coping. J Health Soc Behav 1978;19:2-21.

**Self-Esteem**

Silber, E & Tippett, JS 1965.  Self-esteem: Clinical assessment and measurement validation.  Psychol Reports, 16, 1017-1071.

**Sexual Functioning Questionnaire**

**Watts, RJ. Sexual functioning, health beliefs, and compliance with high blood pressure medications.** Nurs Res. 1982 Sep-Oct;31(5):278-83.

**STAXI – State-Trait Anxiety Inventory**

Spielberger, C. D., Gorsuch, R. L."> Lushene, R., Vagg, P. R., & Jacobs, G. A. (1983). *Manual for the State-Trait Anxiety Inventory*. Palo Alto, CA: Consulting Psychologists Press.

**SCL-90**

L R Derogatis, K Rickels, A F Rock

The SCL-90 and the MMPI: a step in the validation of a new self-report scale. The British Journal of Psychiatry Mar 1976, 128 (3) 280-289; **DOI:** 10.1192/bjp.128.3.280

**Attitudes Toward Aging**

Patsdaughter, C. A., & Killien, M. Developmental transitions in adulthood: mother-daughter relationships. *Holist Nurs Pract, 1990, 4*(3), 37-46.

**APPENDIX F**

**Sample Daily Diary**


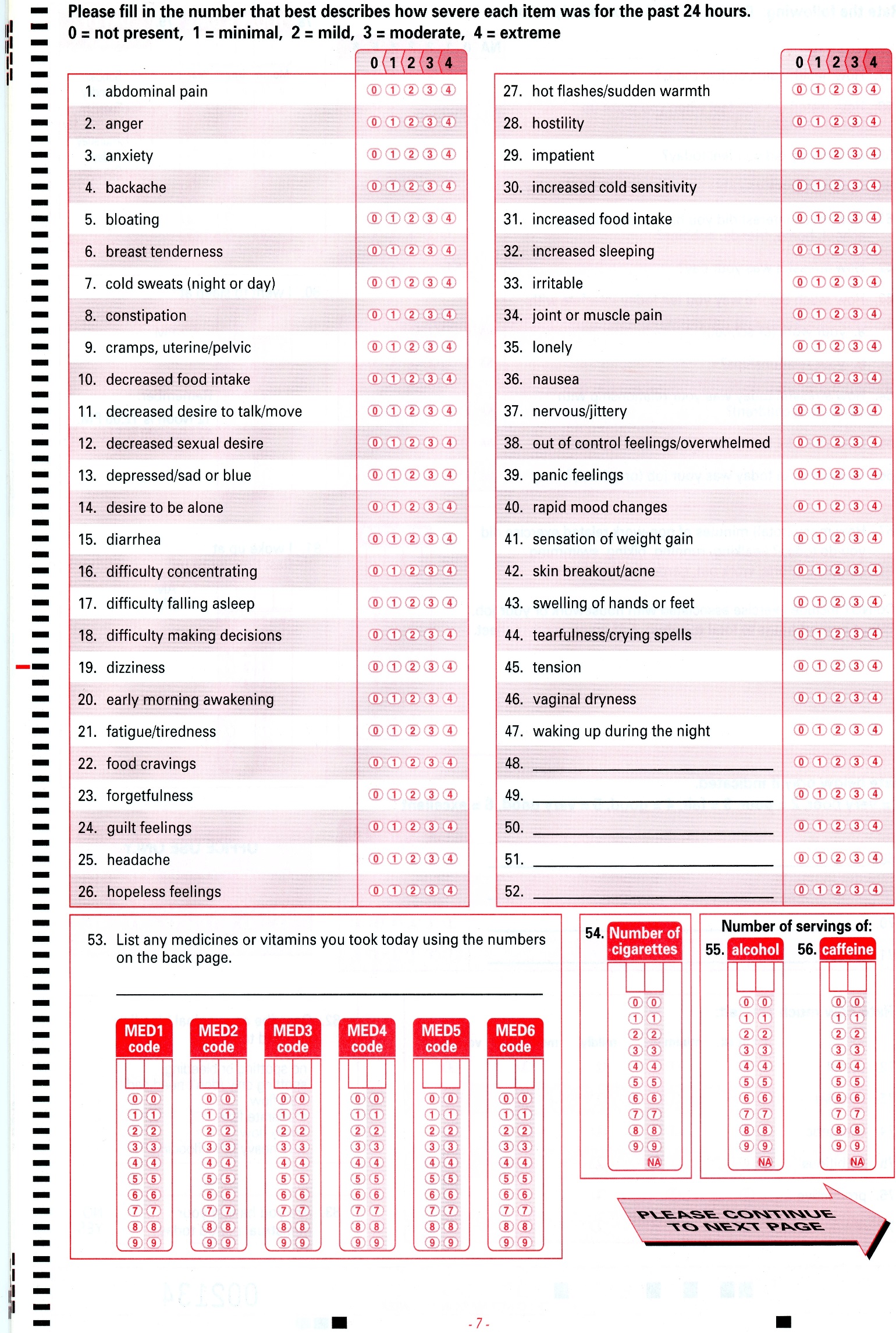


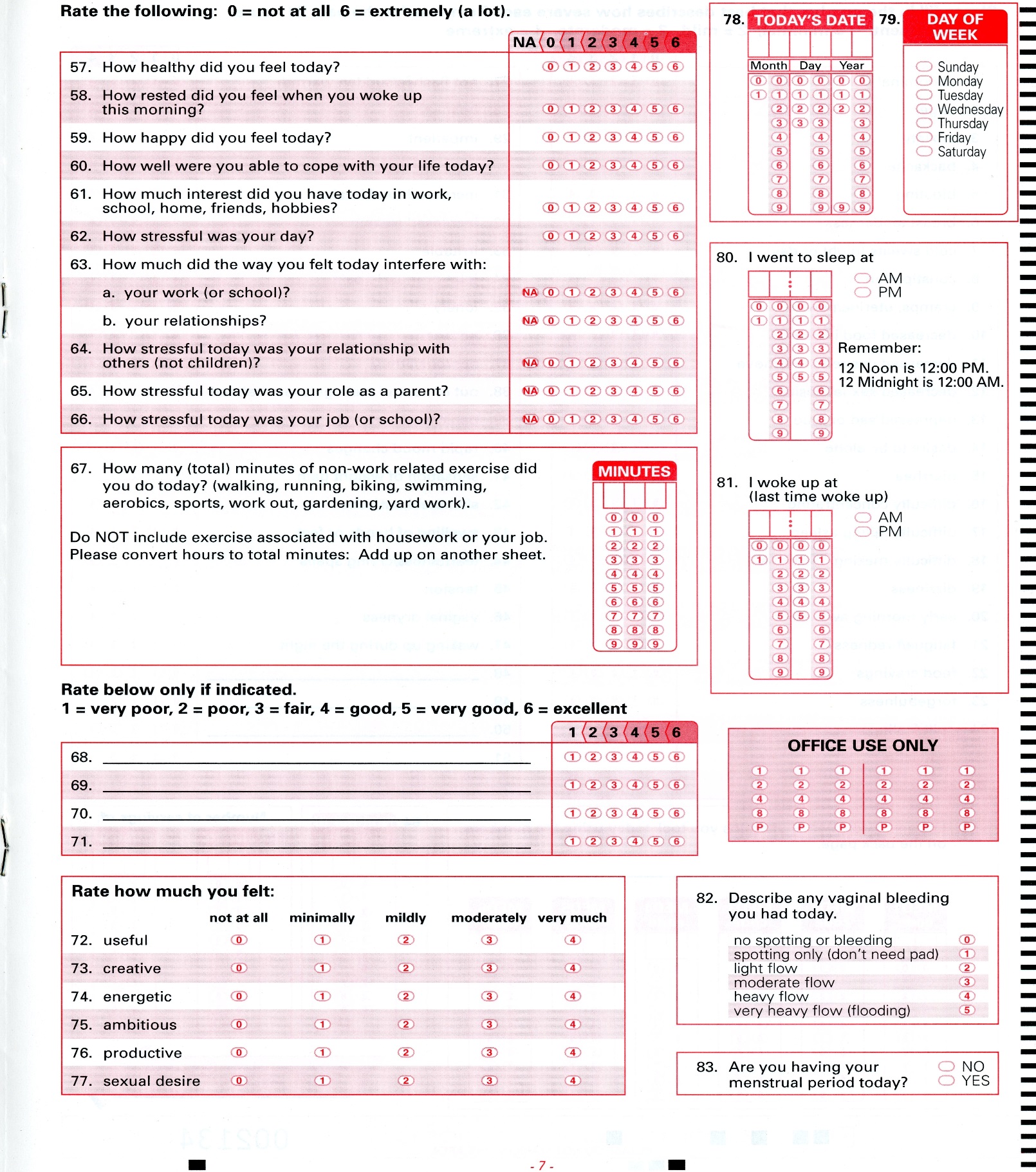


**APPENDIX G**

# **Instructions for Collection of Buccal Cells for Mailing**

• Rinse mouth out with water if you have had a beverage or eaten within 30 minutes of collecting the sample, otherwise, it is not necessary to rinse with water prior to collection.

• Take the brush out of the tube

• Using a slow twisting motion, the brush is rubbed five times in an upward motion on the inside of one cheek and five times in a downward motion on the opposite cheek, then one time across the inside of the upper lip and one time across the inside of the lower lip.

• When collecting from inside the cheek cover as much of the inside surface area as possible. For example, if beginning from the top of the cheek, with mouth opened widely, begin twisting in a downward motion from the bottom of the top teeth to the top of the bottom teeth. Use moderate pressure when collecting sample, it is not necessary to press vigorously.

• Twisting the brush on the inside of the cheek surface helps to accumulate cells on the brush. It is not necessary to twist the brush across the inside of the upper and lower lip, rather just sweep the brush along the inside surfaces of the lips.

• Place the brush back into the tube and place the lid on tightly. Write the date that you collected the sample on the outside label of the tube. Your ID number and study initials will already be on the tube label.

• If you have any questions regarding proper collection technique, contact ______ at (206) 543-4090 (voice mail box #1).

**APPENDIX H**

**GENOTYPING SEQUENCING**

**PCR Primers and Allele-Specific Probes Used in the Genotyping Assays**

| **Polymorphism** | **Forward (FP) and Reverse (RP) PCR Primers** | **Wild-Type and Variant Allele Probes** |
| --- | --- | --- |

ESR1 *XbaI* (FP) 5'-gacacatgttctgtgttgtccatca-3’ NA

(rs9340799)

ESR1 *PvuII* (RP) 5'-gctggtttctaatagacttaatgtttttg-3' NA

(rs52234693)

CYP17 A1/A2 (FP) 5’-gccctttaaaaggcctccttgt-3’ 5’FAM-tcttctactccactgctgtctatct-3’

(rs743572) (RP) 5’-ccacgagctcccacatggt-3’ 5’TET-cttctactccaccgctgtctatct-3’

CYP19 3’UTR (FP) 5’-cccaagaaactcagacaggtgtct-3’ 5’FAM-cagtacccactctgga-3’

(rs10046) (RP) 5’-caaggatggatgatttgtatgtgaac-3’ 5’VIC-tcagtacctactctggagc-3’

HSDB1 (FP) 5’ gcgtgatgaaatcaagaacctcaaa 3’ 5’-VIC-ccccagt**c**cagccc-mgbnfq-3’

rs2830 (RP) 5’ tgcagccgggcatgag 3’ 5’-FAM-cccagt**a**cagccc-mgbnfq-3’

(92 bp)

HSDB1 (FP) 5’ gcgtgatgaaatcaagaacctcaaa 3’ 5’-VIC-ccccagt**c**cagccc-mgbnfq-3’

rs615942 (RP) 5’ tgcagccgggcatgag 3’ 5’-FAM-cccagt**a**cagccc-mgbnfq-3’

(87 bp)

HSDB1 (FP) 5’ cacctaactcctcctgcagatg 3’ 5’-VIC-ccccttgg**g**tatcca-mgbnfq-3’

rs592389 (RP) 5’ gtccatattaaaggccagacacagt 3’ 5’-FAM-ccccttgg**t**tatcca-mgbnfq-3’

(65 bp)

CYP1A1*2C (FP) 5’-gcatgggcaagcggaagt-3’ 5’FAM-cggtgagaccattgcccgct-3’

(rs1048943) (RP) 5’-cacccgttgcagcaggat-3’ 5’FAM-cagcgggcaatgttctcaccg-3’

5’TET-cagcgggcaacggtctcacc-3’

5’TET-agcgggcaacgttctcaccg-3’

CYP1B1 Ala^119^Ser (FP) 5’-caggccctggtgcagca-3’ 5’FAM-ccggccggccttcgc-3’

(rs1056827) (RP) 5’-agtggccgaaagccatgct-3’ 5’TET-accggccgtccttcgcc-3’

CYP1B1 Leu^432^Val (FP) 5’-cccaaggacactgtggtttttgt-3’ 5’FAM-tgaatcatgacccagtgaagtggccta-3’

(rs1056836) (RP) 5’-tgttgatgaggccgtccttgt-3’ 5’TET-tgaatcatgacccactgaagtggccta-3’

CYP19 (TTTA)n (FP) 5’-ctctggaaaacaactcgaccct-3’ NA

(rs2389) (RP) 5’-ggttacagtgagccaaggtcgt-3’ NA

VIC = Applied Biosystems Inc. Trademark

FAM = 6-carboxy-fluorescein

TET = 6-carboxy-1,4-dichloro-2’,7’-dichloro-fluorescein

MGB NFQ = Minor Groove Binder Nonfluorescent Quencher

**APPENDIX I**

**Multilevel Multivariate Modeling Procedure**

Mixed effects modeling using the R library36–40 was used to test a model (Figure 1) to determine whether age, MT stage, factors related to the MT, stress-related factors, social factors, symptoms, and health-related factors were significant predictors of each of the cognitive symptoms over time. Age was centered at the group mean to enable the interpretation of the effect of age on each cognitive symptom.

The first stage tested age alone as a predictor of each of the cognitive symptoms. Using difficulty concentrating as an example, the first model postulated that overall levels of difficulty concentrating could differ from woman to woman (random intercept), but the scores would change with age in a common manner (fixed slope).

The second stage extended the first to postulate that each woman had a different mean level of difficulty concentrating and rate of change (random intercept, random slope). The best fitting model (fixed or random slope) was assessed by using maximum likelihood estimation with Akaike Information Criterion (AIC)40. When the best fitting model was found, that model was extended by adding covariates iteratively to test the effect on cognitive symptoms over time.

For the third stage, all covariates that significantly improved the model fit to the data when entered individually were added simultaneously into a final multivariate model for each of the cognitive symptoms. Finally, a reduced final model was considered using only the significant variables from the final model. Because these analyses were used as a basis for explanation and to stimulate further mechanistic studies, a p value of 0.05 was used as the criterion for significance. Different numbers of women and observations occurred with each variable tested because the analysis required pairing of observations of the outcome and predictor variables at each time point. A description of the mathematical model which provides the foundation for the analytic approach is described below.

**Mathematical Model for Age-based MLM Analyses**

**(CES-D scores used as an example from Menopause, 2008, vol. 15, no. 2, pp. 232)**

Let y_ij_ represent the jth CES-D score obtained from the ith woman, where i = 1, …, M and j = 1, …, n_i_. Here M = 302 is the total number of women who have at least one CES-D score in one of the four stages, and n_i_ is the total number of CES-D scores for the ith woman (whereas n_i_ ≥ 1 for all i, the value of n_i_ varies from woman to woman). Let x_ij_ represent the corresponding age for the woman when the value y_ij_ was recorded. In the models below, x_ij_ is centered at 46.5, which is the approximate sample mean of all recorded ages.

The first age-based model assumes that

y_ij_ = β_1_+ b_1;i +_ β_2_ (x_ij_-46:5) + ε_ij,_ (1)

where the fixed effect β_1_ represents the mean CES-D score at age 46.5 over the population of women; b_1;i_ is a random variable (RV) that is normally distributed with mean zero and variance σ^2^ _1_ (this RV represents the deviation from β_1_ for the ith woman), β_2_ is a fixed slope, and ε_ij_ represents the error terms, which are independent and normally distributed with mean 0 and variance σ^2^ε (the RVs b_1,i_ and ε_ij_ are assumed to be independent of each other). This model basically postulates that whereas the overall levels of CES-D scores can differ from woman to woman, the scores change with age in a common manner.

The second model differs from the first in that it postulates a random slope for each woman:

y_ij_ = β_1_ + b_1;i_ + b_2;i_ (x_ij_-46:5) + ε_ij,_ (2)

where β_1_, b_1;i_ and ε_ij_ are interpreted in the same manner as the first model, whereas b_2,i_ is an RV representing the slope associated with the ith woman. Assume that b_2,i_ is normally distributed with mean β_2_ and variance σ^2^ _2_. The RVs b_1,i_ and ε_ij_ are assumed to be independent of each other, as are b_2,i_ and ε_ij_; however, b_1,i_ and b_2,i_ are allowed to be correlated. This model basically postulates both the overall levels of CES-D scores and their slopes can differ from woman to woman.

The third model extends the second by adding one or more covariates. In the case of a single covariate z_ij_, the model takes the form

y_ij_ = β_1_ + b_1;i_ + b_2;i_ (x_ij_-46:5) + β_3,i_ z_ij_ + ε_ij,_ (3)

where β_3,i_ is a fixed effect associated with z_ij_. Additional covariates are added in an obvious way.
